# Mechatronic redesign of mechanical brushing apparatus to evaluate abrasive wear of road construction materials

**DOI:** 10.1016/j.mex.2025.103425

**Published:** 2025-06-11

**Authors:** Nathalia Marín-Pareja, Camilo Rodríguez, Eliana Llano, Gloria Restrepo

**Affiliations:** Faculty of Engineering, Applied Physicochemical Processes Group, Universidad de Antioquia, Colombia

**Keywords:** Wear testing, Tribology, Mechatronic integration, Civil infrastructure, Nigel Cross’s methodology, Abrasive wear testing of road construction materials using a laboratory-scale mechanical brushing apparatus

## Abstract

Wear resistance is a fundamental mechanical property of materials. In road construction, the abrasive wear of materials leads to the deterioration of road surfaces, reducing the lifespan of civil infrastructure. Thus, materials used for road construction must resist wear caused by friction with vehicle tires and environmental factors that contribute to material degradation. Our goal was to optimize the current abrasive wear test by enhancing both the structural configuration and the performance of the existing mechanical brushing apparatus.

• The brush used in the original mechanical equipment was replaced with a new wear system configuration that allows for the interchangeability of elements responsible for exerting abrasion forces. Hence, the test provides a more reliable assessment of material abrasive wear and offers repeatable and reproducible results.
• By integrating mechanical design, modern control instrumentation, and user interfaces, this study presents an advanced application of mechatronics in laboratory equipment to improve user interaction, testing accuracy, and reliability.
• The development of versatile laboratory equipment to measure the performance parameters of materials contributes to the standardization of tests that explain the behaviors of these materials, which are critical for both academic researchers and industry professionals involved in civil infrastructure maintenance.

## Graphical abstract

**Figure:**
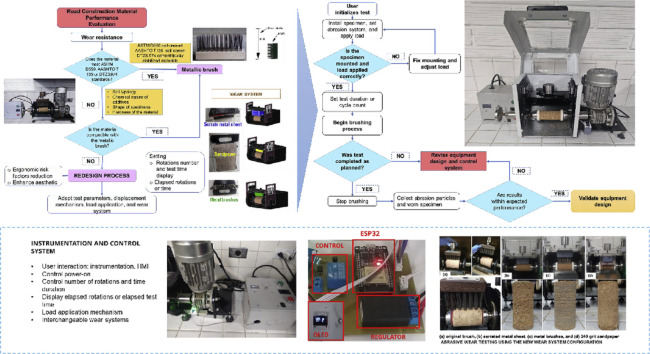

Specifications table

**Table:** 

Subject area:	Engineering
More specific subject area:	Materials science, Civil Engineering, mechatronic, tribology
Name of your method:	Abrasive wear testing of road construction materials using a laboratory-scale mechanical brushing apparatus
Name and reference of original method:	Standard Method of Test for Wetting-and-Drying Test of Compacted Soil-Cement Mixtures. Standard by American Association of State Highway and Transportation Officials, AASHTO T135, 2022 [6]. Standard Test Methods for Wetting and Drying Compacted Soil-Cement Mixtures. Standard by American Society for Testing and Materials, ASTM D559, 2015 [7]. Test method - Determination of the wet-dry durability of compacted and cured specimens of cementitiously stabilized materials by mechanical brushing. Standard by Tanzania Bureau of Standard, DTZ 974, 2021 [13].
Resource availability:	Not Applicable

## Background

**Fig. 1:**
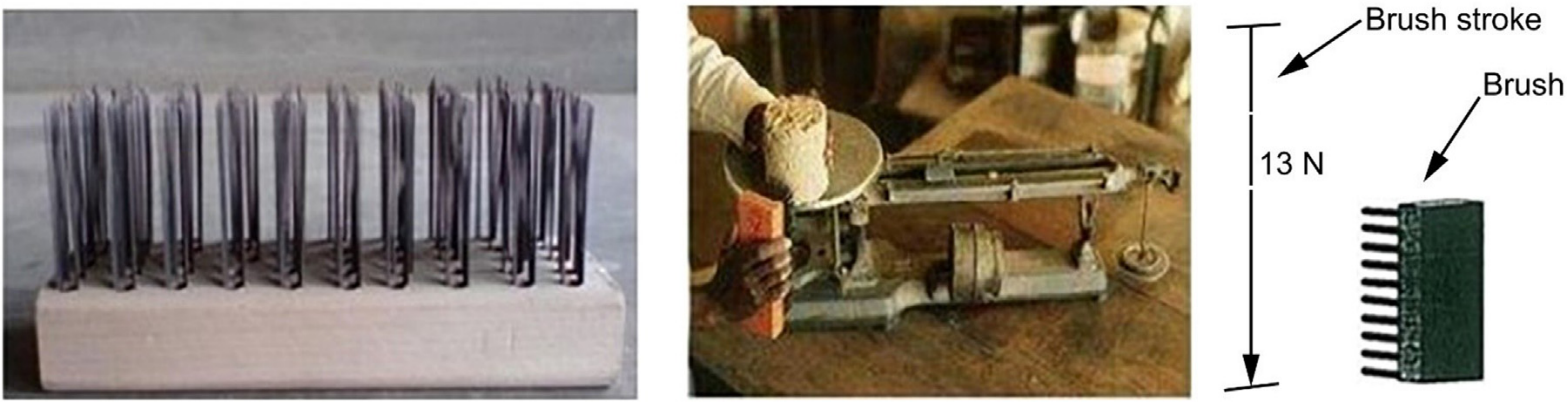
Hand brush to wet/dry durability test (adapted from [5,7,11]).

From a tribological perspective, surface wear is defined as the mechanical degradation that gradually removes material from one or both interacting solid surfaces in relative motion [1]. Abrasive wear occurs when particles come into contact with and wear away softer surfaces, either by cutting or scraping. Wear can be quantitatively assessed by measuring the mass, volume, or height of material removed per unit time or sliding distance [2]. In road construction, abrasive wear is generally induced by hydraulic or mechanical action from traffic loads, which deteriorates the road surface via the gradual loss of surface particles [3,4]. Various methods have been established to evaluate abrasive wear. These methods are based on the use of a brush that is manually passed over a test specimen to erode the surface of the sample (Fig. 1) [[5], [6], [7]]. Different studies aimed at standardizing laboratory procedures have shown that, despite the simplicity of the tests employed, brushing action exhibits inadequate repeatability and reproducibility, owing to the susceptibility of the manual technique [[8], [9], [10], [11], [12]].

**Fig. 2:**
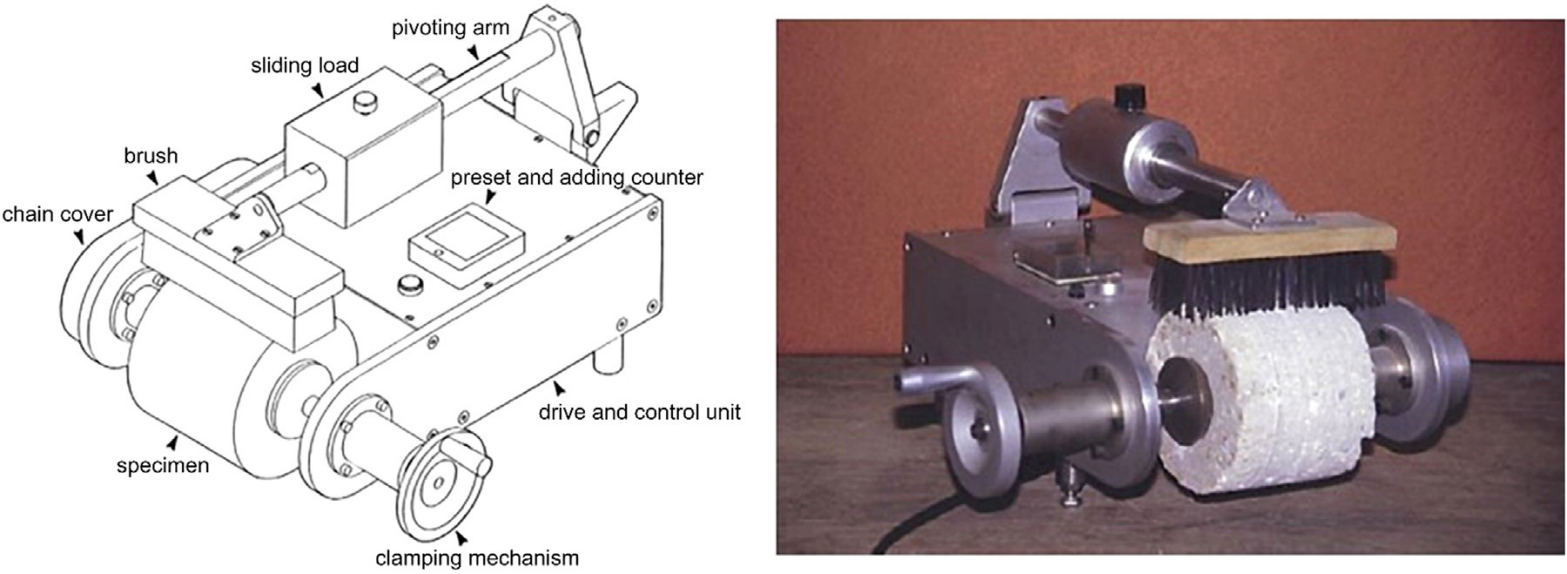
Mechanical brushing apparatus (adapted from [3,13]).

In South Africa, after experimenting with various techniques, the Tanzania Bureau of Standards adopted a mechanical brushing technique to overcome the problems of repeatability and reproducibility associated with the manual brushing process [9,13] (Fig. 2).

**Fig. 3:**
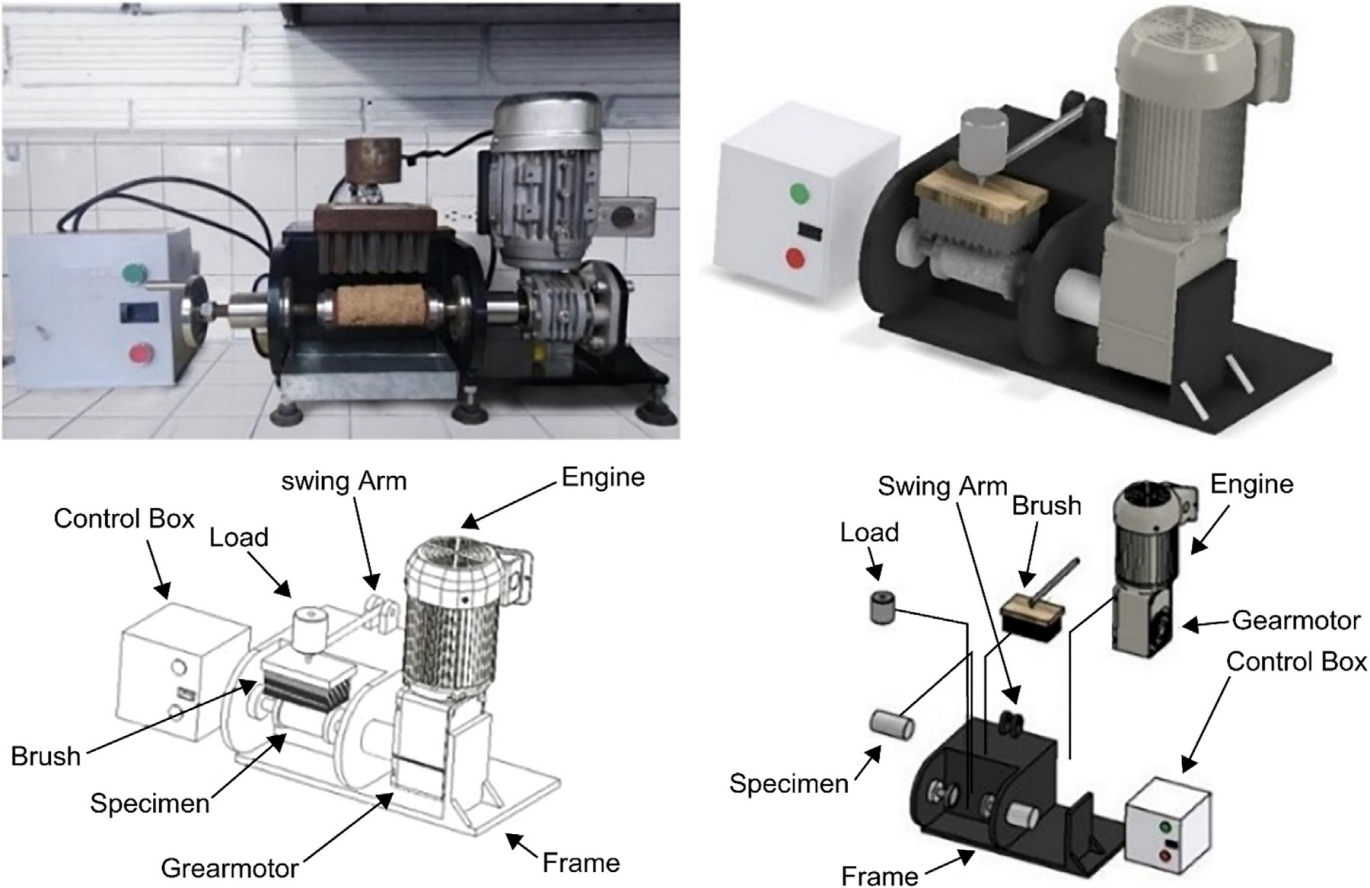
Current mechanical brushing apparatus.

Using this model as a basis, the authors designed and constructed a mechanical brushing apparatus to evaluate the abrasive wear of road construction materials (Fig. 3).

The implemented wear test induces material loss from the surface of a specimen that rotates around its longitudinal axis under the action of a brush loaded with a specific mechanical force. The brushing includes an electronic device to control the number of revolutions and duration.

Following years of operation, it was determined that the brushing test had limitations when evaluating gravelly soils and soils treated with different materials, which restricted its application in the intended field. The type, size, and shape of the brush, including the abrasion mechanism during the test, were not suited to different soil typologies or different additives used as soil stabilizers. The wear induced by the brush was not uniform across the entire surface of the sample, indicating that the test results were neither repeatable nor reproducible. Consequently, it has been challenging to establish a reliable wear test that reflects the wear resistance of road construction materials [14]. Moreover, a comprehensive evaluation of the equipment performance also highlighted the need for improvements in structural, ergonomic, safety, and aesthetic aspects.

The objectives of this work were to overcome the identified technical and operational limitations of the mechanical brushing apparatus by enhancing its structural configuration and performance, as well as to optimize an existing wear test. These enhancements aim to ensure that the wear test results are reliable, repeatable, and reproducible and that the test more accurately simulates field conditions for evaluating the abrasive wear of road construction materials.

## Method details

Modifications to the current brushing apparatus were needed to address the identified limitations in its functionality, design, and operation. These changes necessitated the application of holistic methodological approaches, such as mechatronics, which is a field in product engineering that synergistically integrates multiple engineering disciplines, including mechanical, electrical, computer, electronics, and control engineering [15]. Additionally, the design and manufacturing of devices and equipment require a thoughtful and systematic approach. Considering the prior analysis, the mechatronic redesign of the current mechanical brushing apparatus was conducted with a focus on user needs and a multidisciplinary perspective, following Nigel Cross’s methodology, which emphasizes products with engineering content [[16], [17], [18]]. For the mechatronic redesign of the existing brushing apparatus, key stages of Nigel Cross’s design methodology were implemented, including defining design objectives, determining function and structure, configuring specific design requirements, and identifying key product features [17,19]. Once the requirements and specific characteristics of the new equipment were established, the design and fabrication of the prototypes were executed. Two prototypes were constructed to validate the mechanical design; an instrumentation and control system and three wear-system configurations were incorporated. Several control tests were conducted based on the number of rotations and the maximum operating time to evaluate the instrumentation and control system. For the implemented wear systems, cylindrical specimens of natural soil were prepared, compacted, and subjected to the wear test.

### Defining design objectives

Based on the established purpose for redesigning the brushing apparatus, the following design objectives were determined:

- Adapting the type and shape of the wear brush to match the soil typology and chemical nature of additives used as soil stabilizers.
- Improving the clamping and displacement mechanisms of the brush, as well as the load application system.
- Controlling and displaying the number of rotations and test time parameters for specimens.
- Modifying the structural configuration of equipment to reduce ergonomic risk factors, ensure user safety, and enhance aesthetic aspects.

**Fig. 4:**
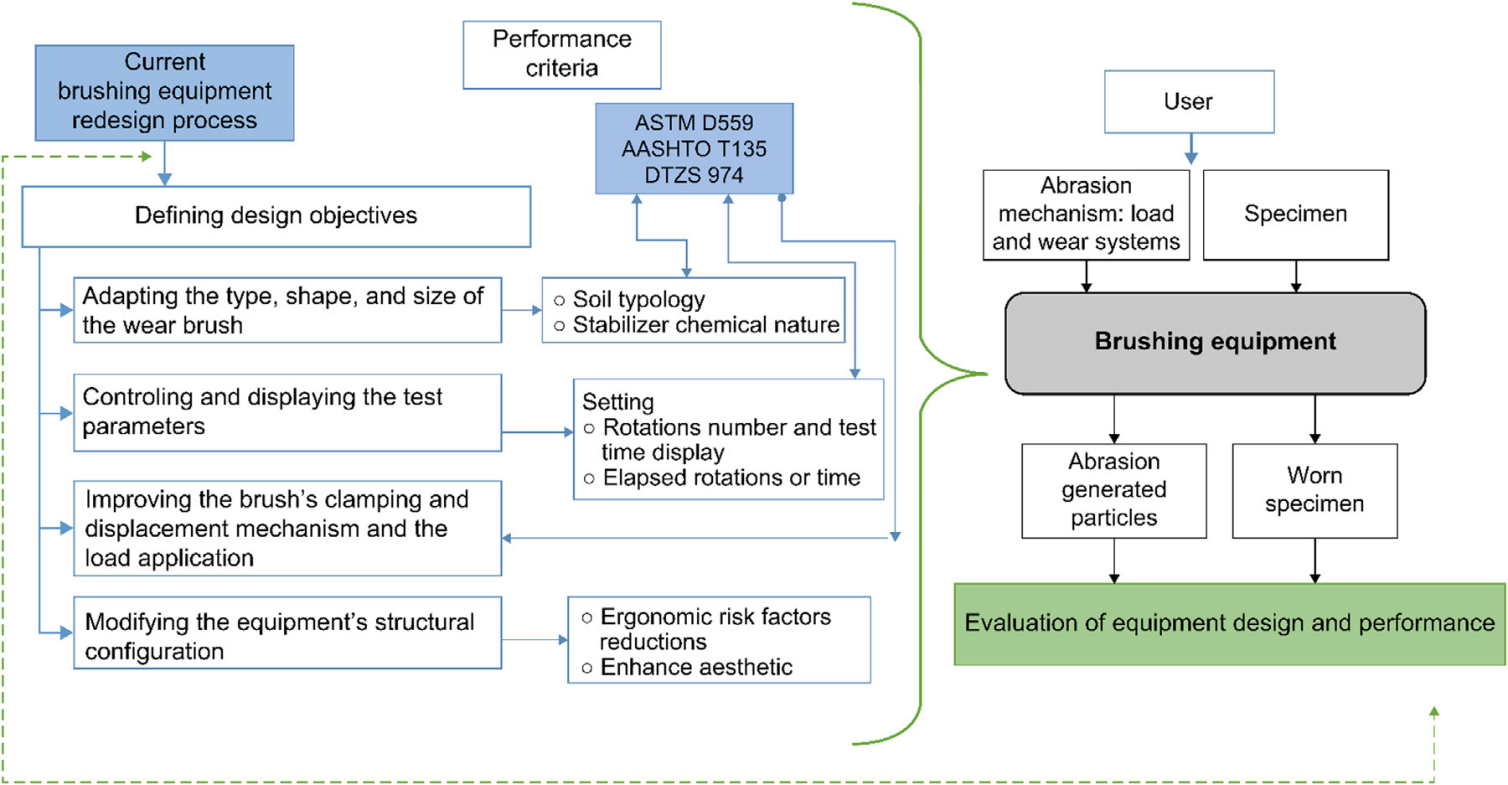
Mechatronic redesign of current brushing apparatus; design objectives.

Fig. 4 presents a diagram that illustrates the design objectives and requirements identified to fulfill the established performance criteria.

### Determining functions and structure

**Fig. 5:**
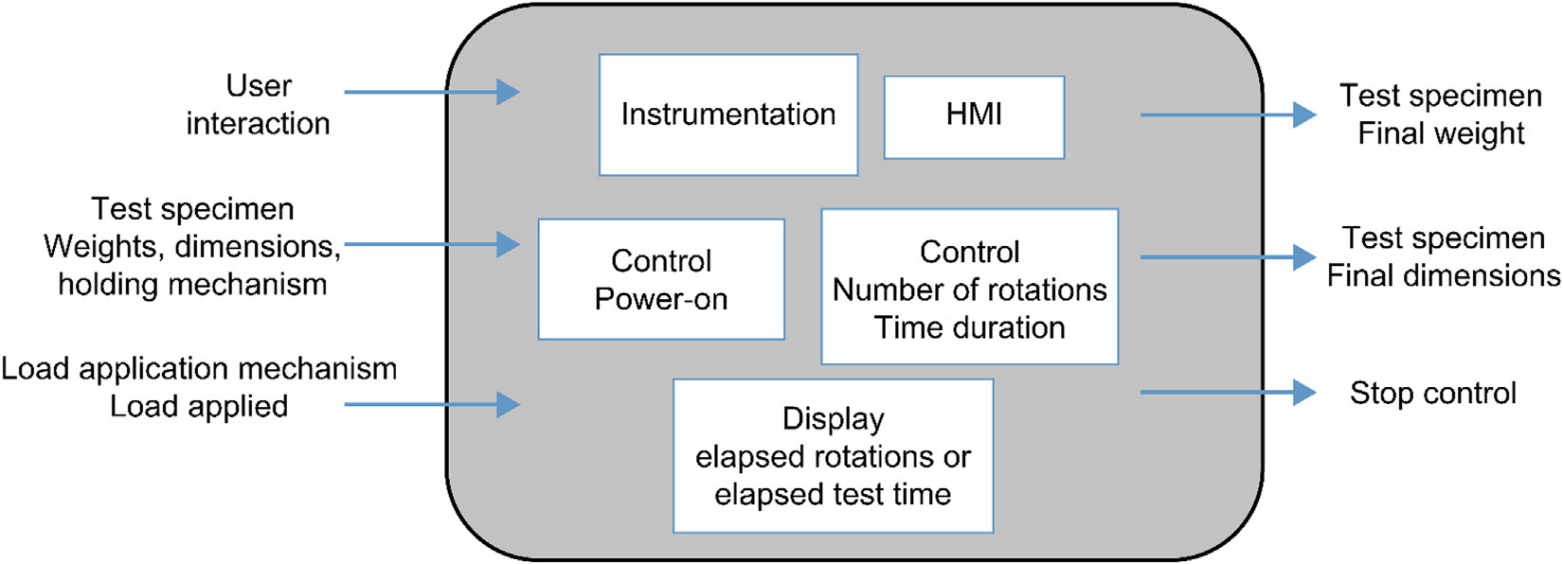
Functions and structure; Niger Cross transparent box model.

In this stage, the main functions that the equipment must fulfill and the structure necessary to support these functions were identified, considering the requirements of the wear test and the materials to be evaluated. Based on these premises and the analysis of the functions using the Cross methodology [17], the input and output variables of the design process were established, as illustrated in Fig. 5.

### Configuring specific design requirements

The specific design requirements considered fundamental to developing a functional brushing apparatus prototype were classified into the components described below:

- Mechanical: material characteristics and general dimensions.
- Operating conditions: general conditions and parameters of the abrasive wear test.
- Electronics and Instrumentation: operating conditions of sensors and actuators; specifications of the essential parts of the human–machine interface (HMI) and the operating code for the microcontroller.

These requirements, aligned with the design objectives, generated the specific characteristics of the prototype components (outlined in the subsequent section).

### Determining key product features

In this stage, the essential attributes and engineering requirements were defined with respect to the operation, performance, and specific use of the equipment, also considering ergonomic, safety, and aesthetic considerations. Employing a trial-and-error methodological strategy, rapid prototyping techniques were utilized using locally available low-cost materials. This approach facilitates quick iteration and validation of ideas, incorporating the design philosophy central to the prototyping culture, which is fundamental to the design thinking process. Additionally, user needs to derive from their experience with testing and operating the existing equipment were considered, as well as regulatory aspects described in AASHTO T135 [6], ASTM D559 [7], and the model proposed by the Tanzania Bureau of Standards DTZS 974 [13]. Table 1 outlines the fundamental elements necessary to develop a functional prototype according to the design requirements and the classification established in the previous section.

**Table 1:** Design requirements.

Component	Description
Mechanical requirements	
Material	Polylactic acid (PLA) and acrylic
	Hot-rolled steel with 3/16″ thickness
Remanufacturing processes	Laser cutting
	3-axis computer numerical control (CNC) machining
	3D printing
Wear system	Wear elements: abrasive paper, serrated metal blade, metal brush
	Dimensions of the frame that holds the different wear elements: 84 × 59 × 65 mm (width, depth, height)
	Frame material that holds the different wear elements: acrylic
	Wear system material: PLA
	User handling: tool-free interchangeable brushes
Load application	Linear direction towards the center of the sample, in the upper quadrant
Dust protection chamber specification	IP5X enclosure protection grade
	Material: acrylic
Operating conditions	
Sample rotation revolutions	60 rpm
Operating temperature	20–25 °C (room temperature)
Type of test	Abrasive wear
Operation time	User-defined
Number of rotations	User-defined
Operating load for test	13 N (3 lbf)
On and off system	User-operated on/off switch
Maximum sample size	Diameter 70 mm, height 100 mm
Working voltage	110–120 V
Electronics and instrumentation requirements	
Power	110 V AC, 0.01 A
Sensor power output	5 V, 0.2 A
Electronic modules	ESP32 microcontroller, organic light-emitting diode (OLED) display, optocoupler, relay
Transducers	Infrared sensor, rotary encoder
Protection	Suppresses current spikes and surges

### Prototype development

#### Wear system

The wear system of the original equipment is based on AASHTO T135 [6], ASTM D559 [7], and DTZS 974 [13]. It contains a metal brush constructed from a 200 mm × 60 mm wooden block with 40 mm long steel bristles that are 1.8 mm wide and have 0.5 mm caliber. These bristles are randomly arranged into 180 groups of six wires (Fig. 3).

**Fig. 6:**
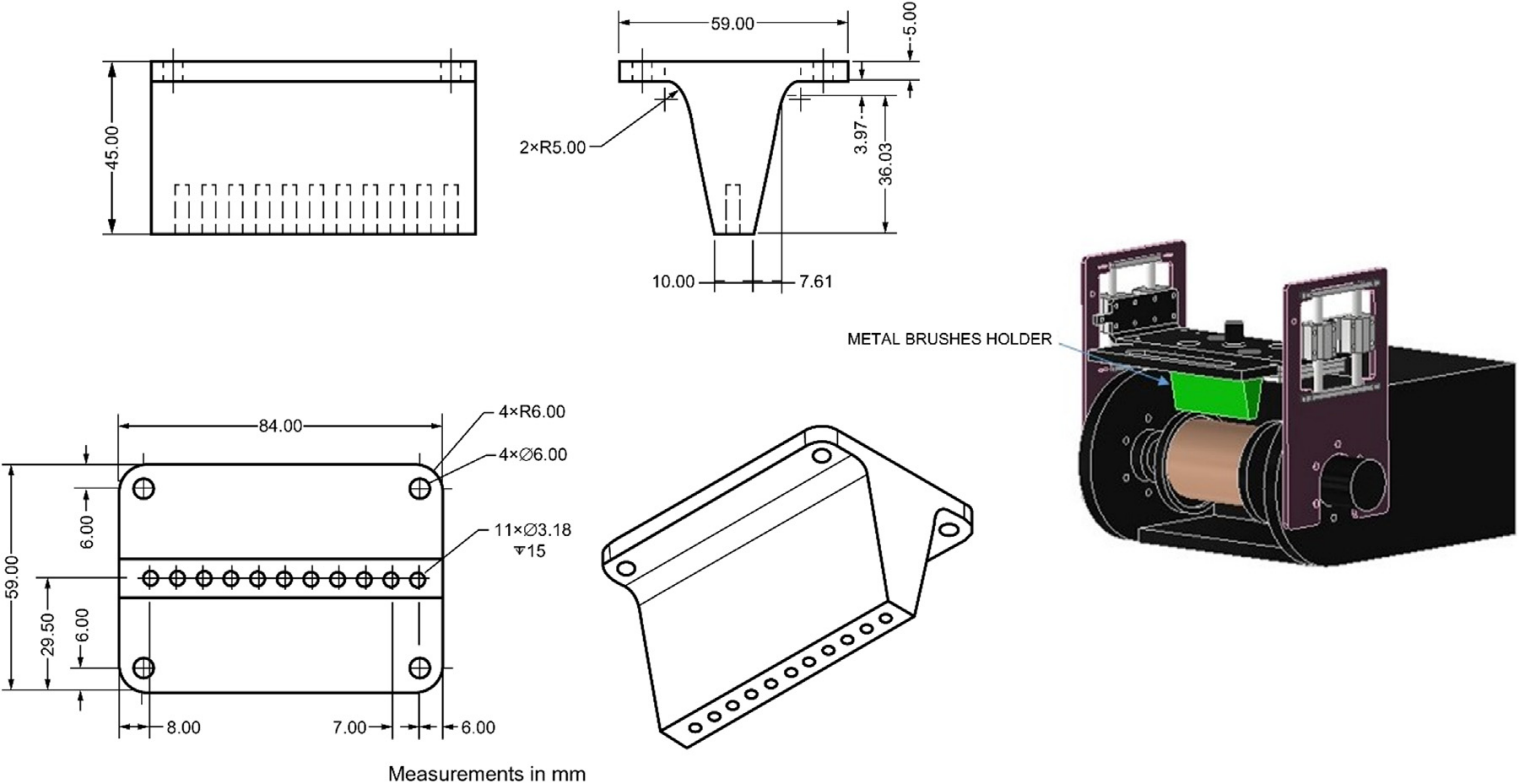
Wear system for installing serrated metal sheet.

**Fig. 7:**
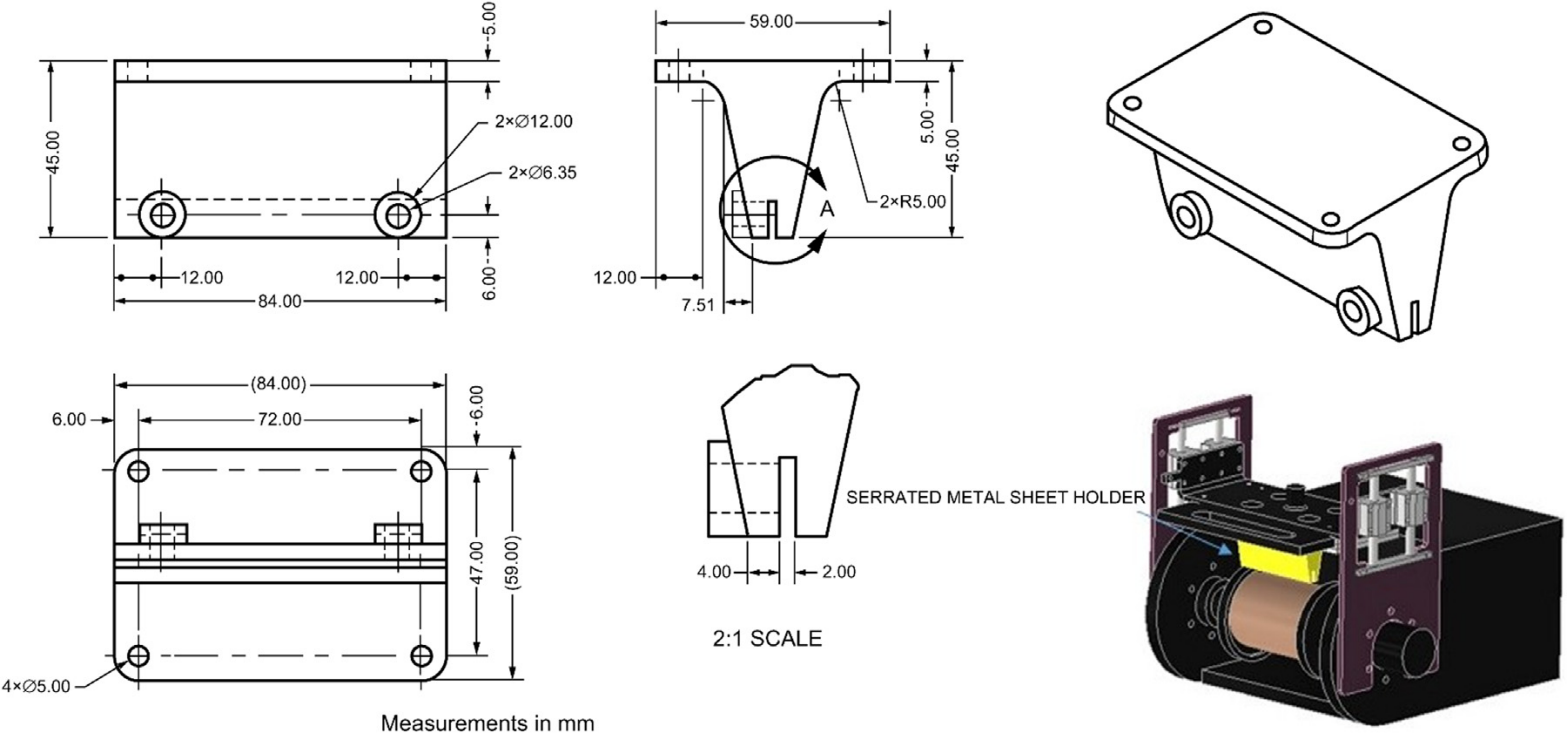
Wear system for installing metal brushes.

**Fig. 8:**
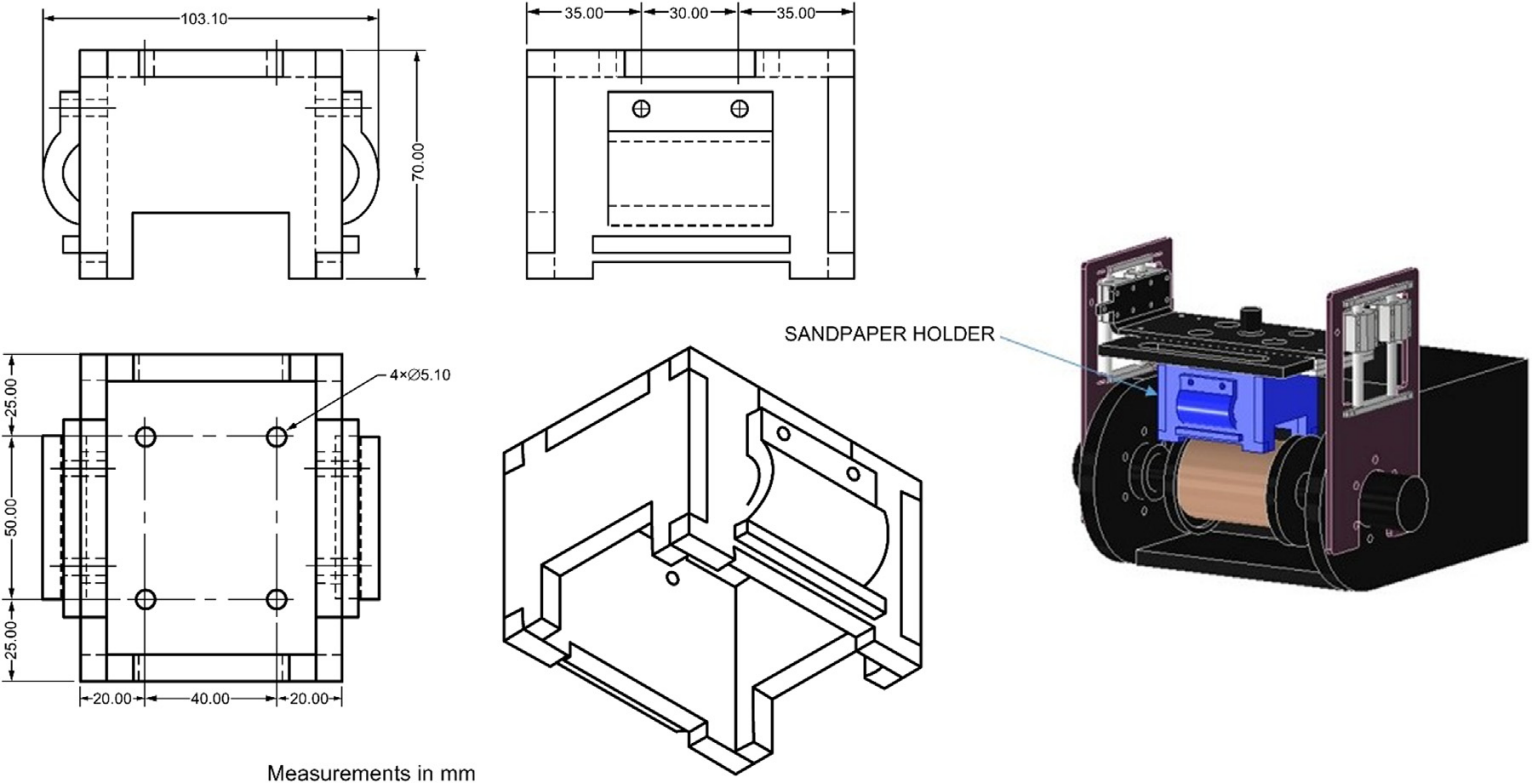
Wear system for installing commercial sandpaper.

To enhance the versatility of the apparatus for abrasive wear testing, a configurable design was conceived to allow for the interchangeability of elements by applying the abrasion force based on the chemical nature of the additives used as soil stabilizers, soil type, and test criteria. Consequently, three wear systems were developed: the first includes a serrated metal sheet for longitudinal wear application on the sample (Fig. 6), the second permits the incorporation of metal brushes (Fig. 7), and the third accommodates commercial sandpaper, offering flexibility based on the degree of abrasion required (Fig. 8). The construction materials used are polylactic acid (PLA) for the serrated metal blade and metal brushes system and acrylic for the abrasive paper system.

#### Wear system clamping and displacement mechanism

**Fig. 9:**
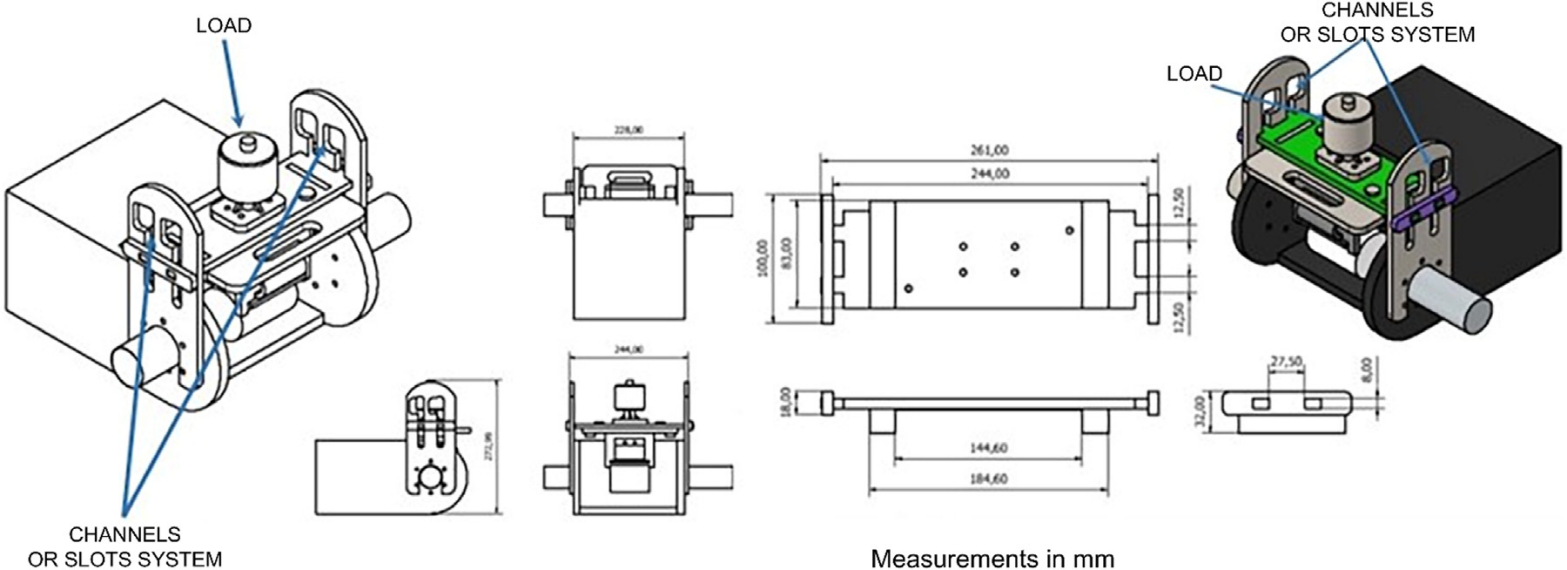
Wear system clamping and displacement mechanism prototype: channels or slots system.

Based on the design requirements, this component must ensure that the load applied to the sample remains constant during the test and that the abrasion force applied by the wear system is homogeneous along the horizontal axis of the specimen. A prototype was developed with a displacement mechanism that uses a system of channels or slots, as depicted in Fig. 9.

**Fig. 10:**
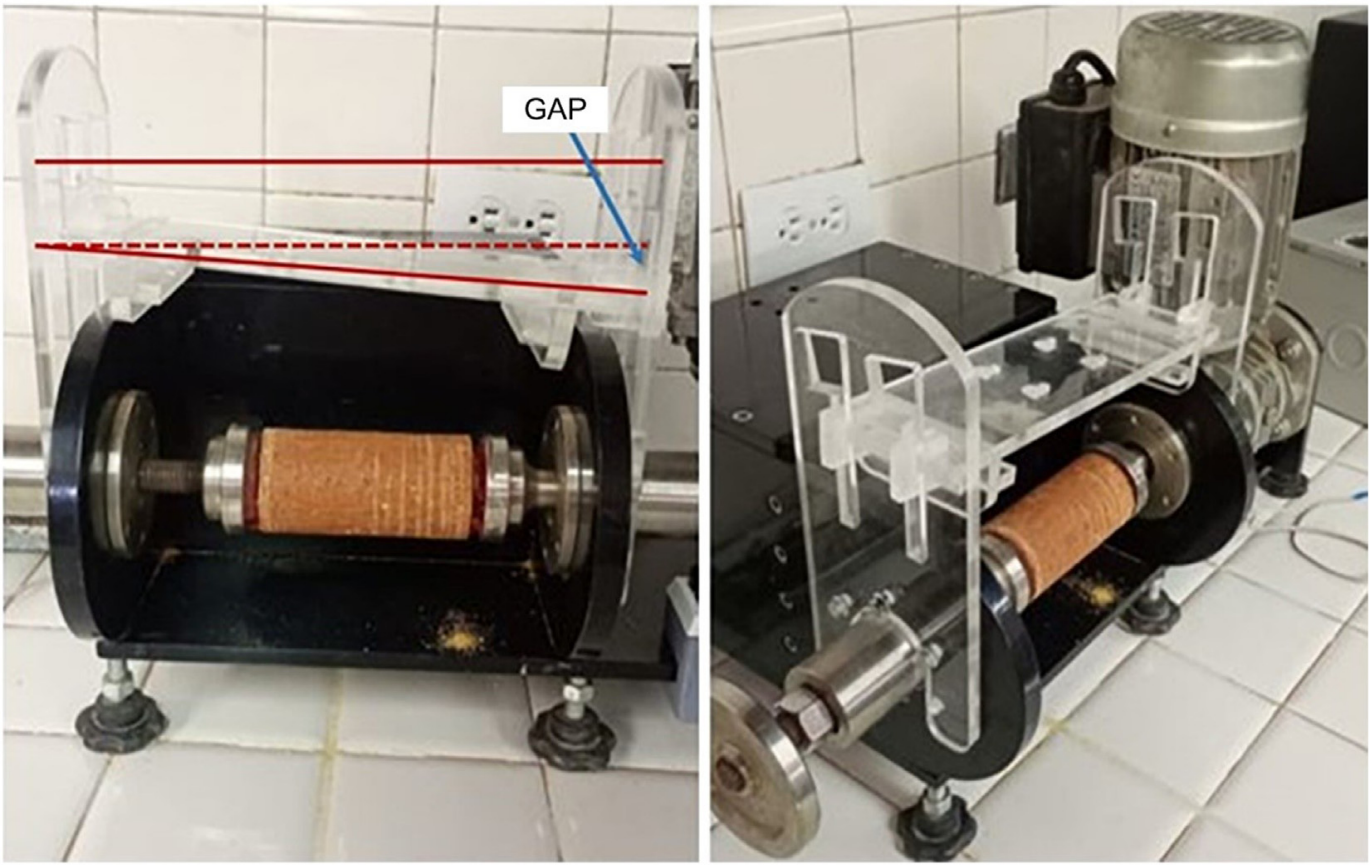
Prototype with nonlinear displacement of the mechanism.

The clamping and displacement mechanism prototype was constructed from acrylic. Upon assembly, the mechanical stability of the equipment was evaluated. A nonlinear displacement of the mechanism was observed, with nonparallel and nonaxial movements toward the center of the sample, as shown in Fig. 10.

**Fig. 11:**
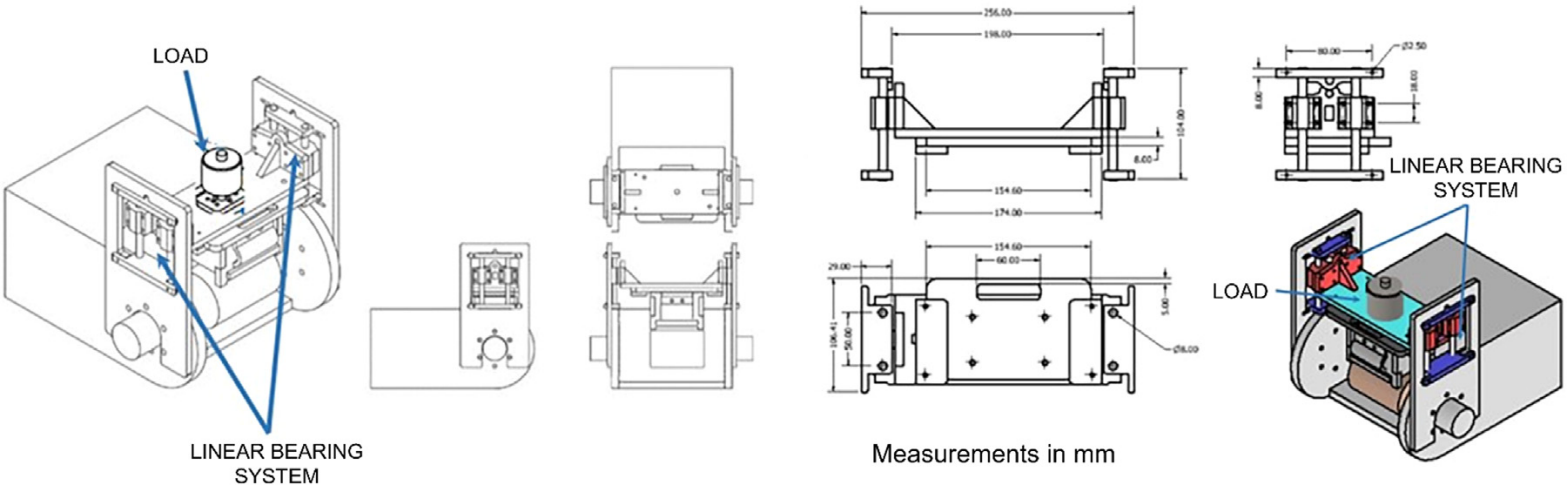
Wear system clamping and displacement mechanism prototype: linear bearing system.

The rapid prototyping and design iterations technique facilitated a computer-aided design (CAD) model adjustment, replacing the displacement using channels or slots with a linear bearing system (Fig. 11).

**Fig. 12:**
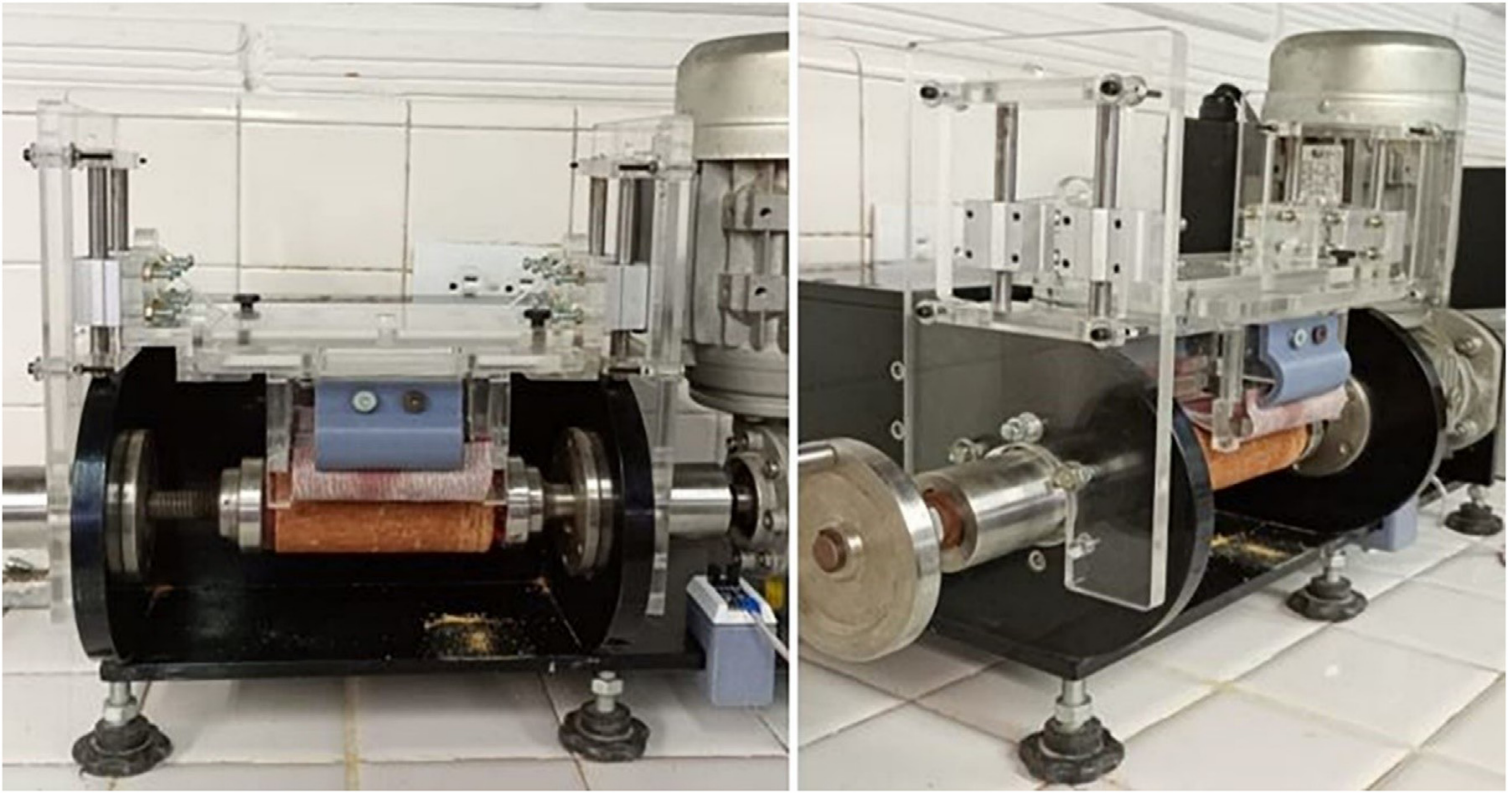
Improved prototype with a linear bearing system.

The wear system clamping and displacement mechanism prototype featuring a linear bearing system was constructed in acrylic with an LM8UU tubular bearing housing made of A380 aluminum casting material. Following several operation cycles, the mechanism maintained its position along the horizontal axis, with smooth movements parallel to the axis. This modification provided mechanical stability to the clamping and displacement mechanism, ensuring that the base plate of the mechanism remained parallel to the surface of the specimen to guarantee symmetrical movement of the wear system and adequate application of the load (Fig. 12).

#### Load application

In the original equipment, the load was applied from a pivoting arm, which moved as the radius of the sample radius decreased during the wear process. In the new configuration, the load remains fixed in the upper quadrant of the wear system’s linear displacement mechanism, ensuring it stays at the same point throughout the test as the sample wears down. In both the current and the redesigned brushing apparatus, the applied load is 13 N (3 lbf) aligns with AASHTO T135 [6] and ASTM D559 [7] standards.

**Fig. 13:**
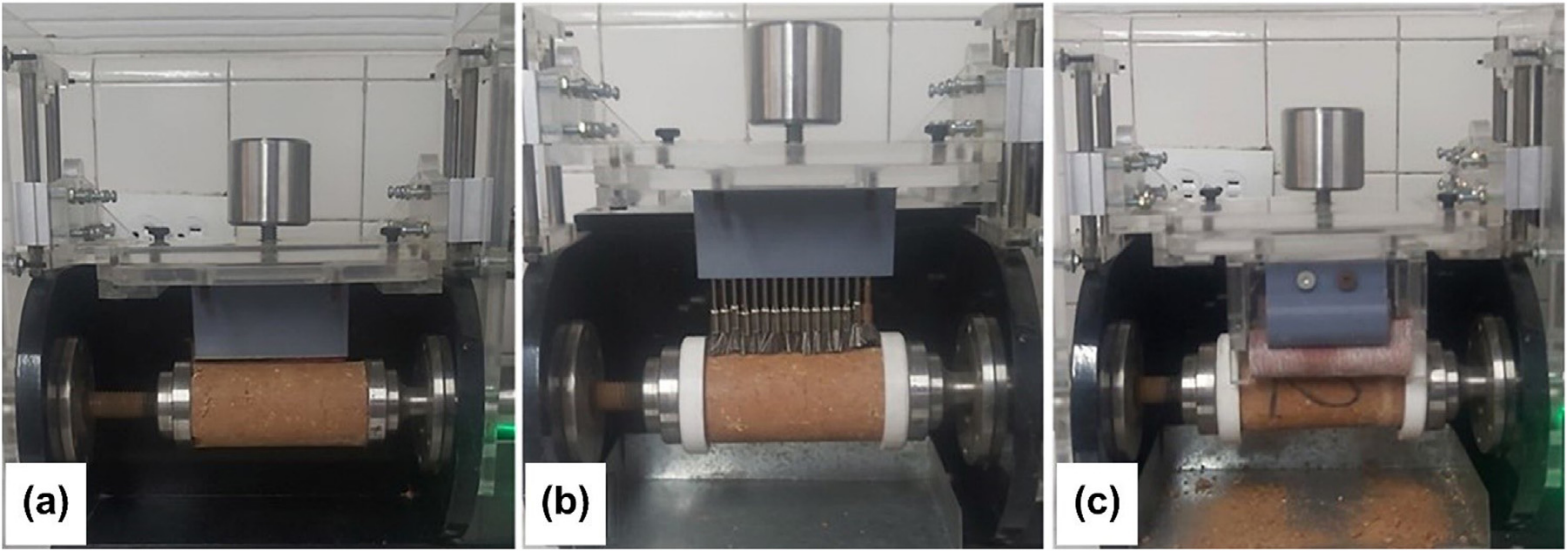
Interchangeable wear systems: (a) serrated metal sheet, (b) metal brushes, and (c) commercial sandpaper.

Fig. 13 shows the three implemented wear systems constructed from PLA material: a serrated metal sheet (Fig. 13a), metal brushes (Fig. 13b), and commercial sandpaper (Fig. 13c). These systems are interchangeable, each designed to securely fit within the clamping and moving mechanism. The load is strategically located in the upper quadrant, integral to the overall functionality of the wear mechanism.

**Fig. 14:**
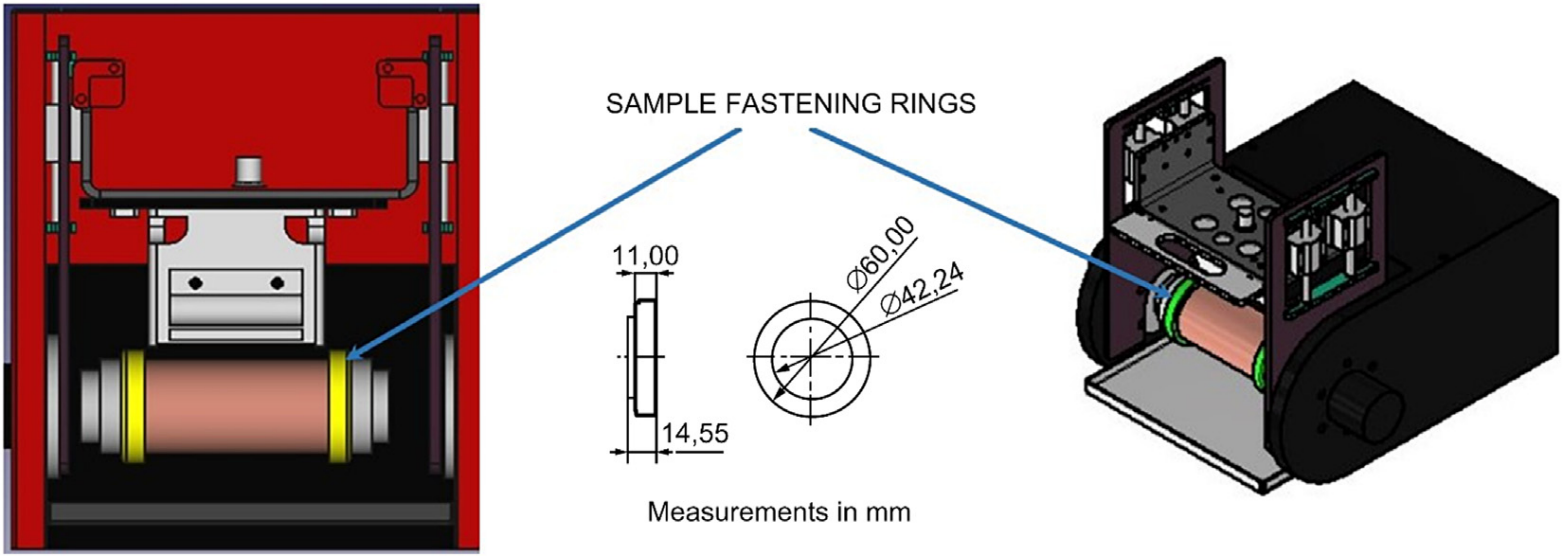
Fastening rings to fix and transmit rotation to the specimen.

Additionally, fastening rings have been designed and fabricated for the specimens to ensure fixation and centering on the rotation axis of the sample, thereby transmitting rotation during the wear test (Fig. 14).

#### Dust protection chamber

The abrasive wear test generates particulate material from the sample surface, posing risks to both the user and the environment. Consequently, a protection chamber against dust emission was designed in accordance with the international dust protection standard, IEC 60,529, achieving a protection degree of IP5X for enclosures exposed to constant dust and humidity conditions [20].

**Fig. 15:**
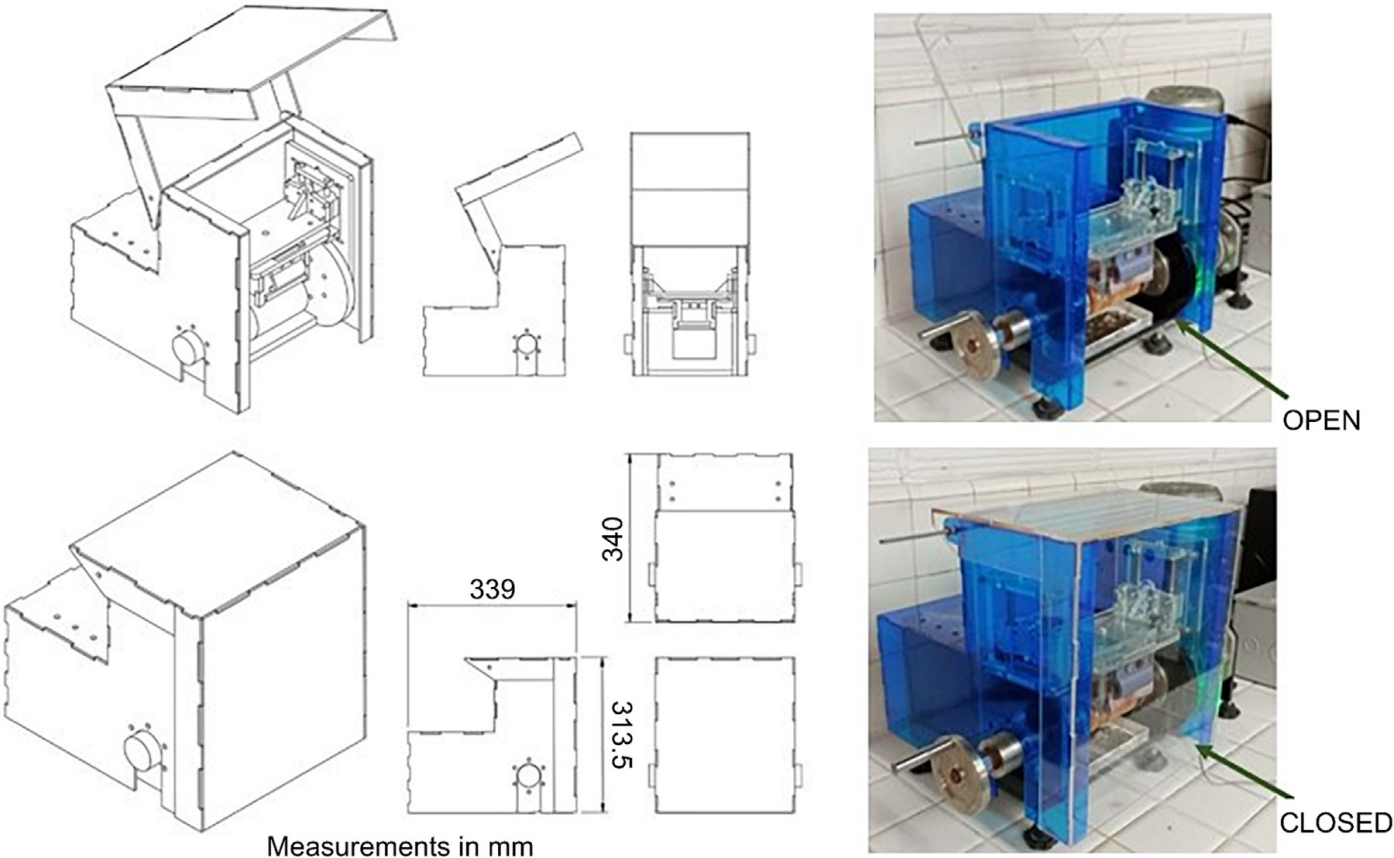
Enclosure CAD model and the constructed protection chamber.

Fig. 15 illustrates the CAD model and the constructed enclosure, which encompasses the wear system, the clamping and displacement mechanism of the brush, and the test specimen. This setup effectively prevents the dispersion of particles generated during the test and their escape to the outside, directing them instead to a collection tray. The enclosure also enhances user safety by shielding against contact with moving parts, preventing potential accidents.

#### Instrumentation and control

**Fig. 16:**
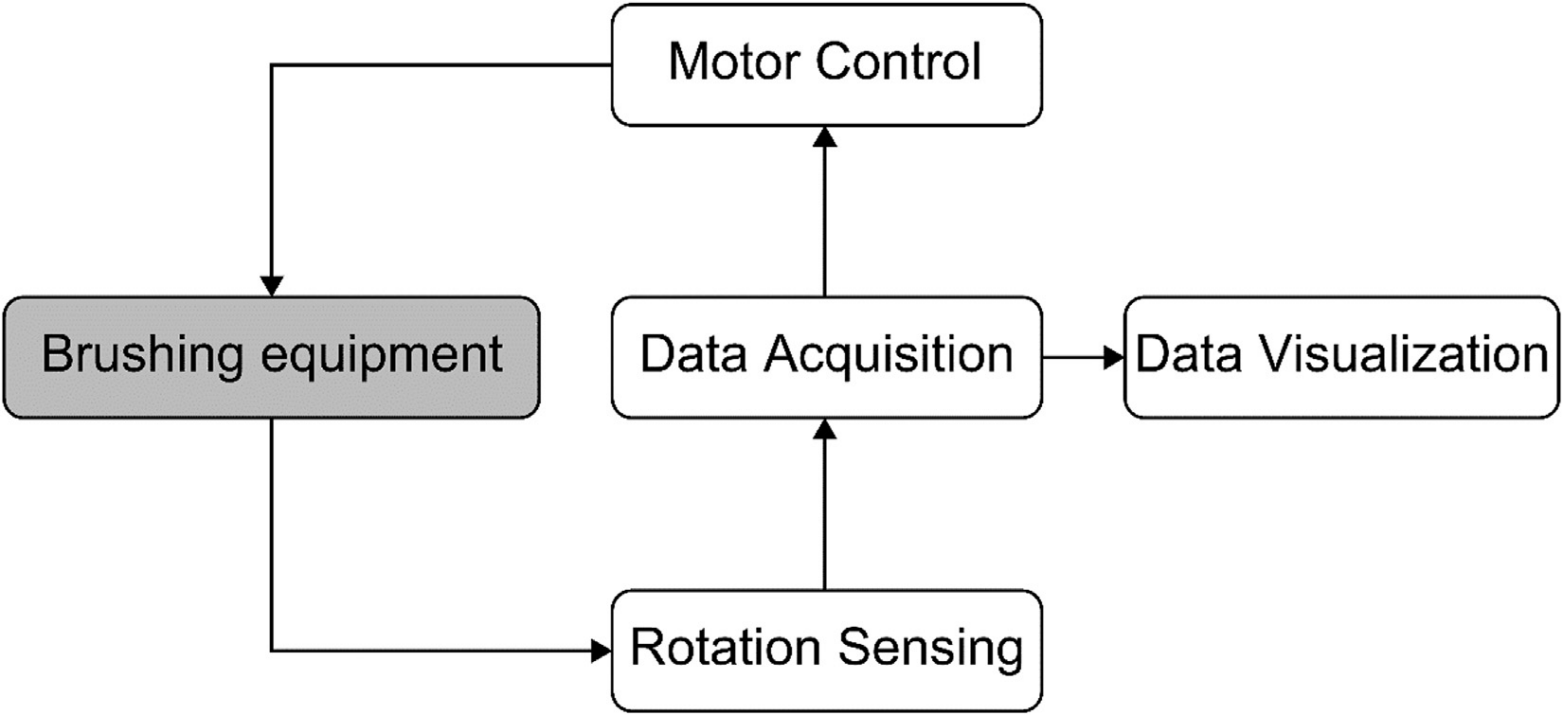
Electronic instrumentation system configuration flowchart.

To fulfill the requirements for setting, controlling, and monitoring critical parameters such as the number of rotations and the test time of the specimen, an electronic instrumentation system was developed. The system comprises subsystems for sensing, motor control, data acquisition, and data visualization [21,22]. Fig. 16 presents the flowchart outlining the configuration of the electronic instrumentation system.

#### Electronics and instrumentation requirements

The low-power consumption 5 V ESP32 microcontroller module was selected for sensor visualization, control, and data acquisition. To accommodate this, a regulatory power supply converting 110 V AC to 5 V DC was incorporated, enabling the electronic board to operate internally at 5 V DC/3 W. A lap counter was integrated to perform user-defined auto-stop functions. The system also includes noninvasive sensing elements such as an infrared sensor and a rotary encoder to navigate the user menu, complemented by an OLED screen for display. Power requirements were determined based on the maximum motor current of 10 A.

#### Motor rotation sensing

**Fig. 17:**
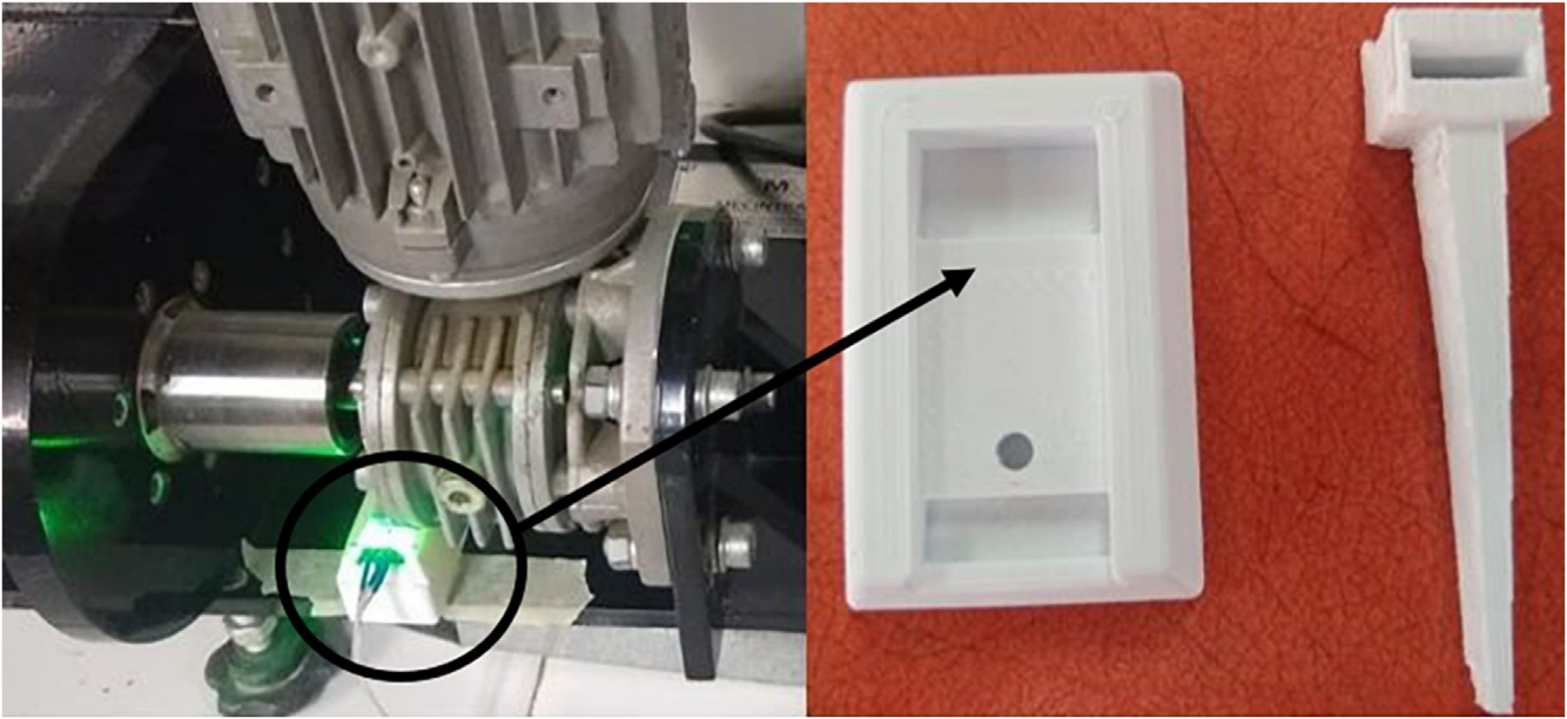
Infrared sensor assembly.

This subsystem is designed to measure the number of motor revolutions. It features an electronic card equipped with an infrared sensor mounted at the base of the shaft, supported by a mechanical pivot marked with a white indicator to detect each rotation [23]. The sensor captures the rotational movement, encoding it into a digital signal with a pulse width of 5 V DC. The signal is then transmitted to the data acquisition system, which tracks the number of rotations and initiates the time counter from the moment the white marker passes over the sensor for the first time (Fig. 17).

#### Motor control

**Fig. 18:**
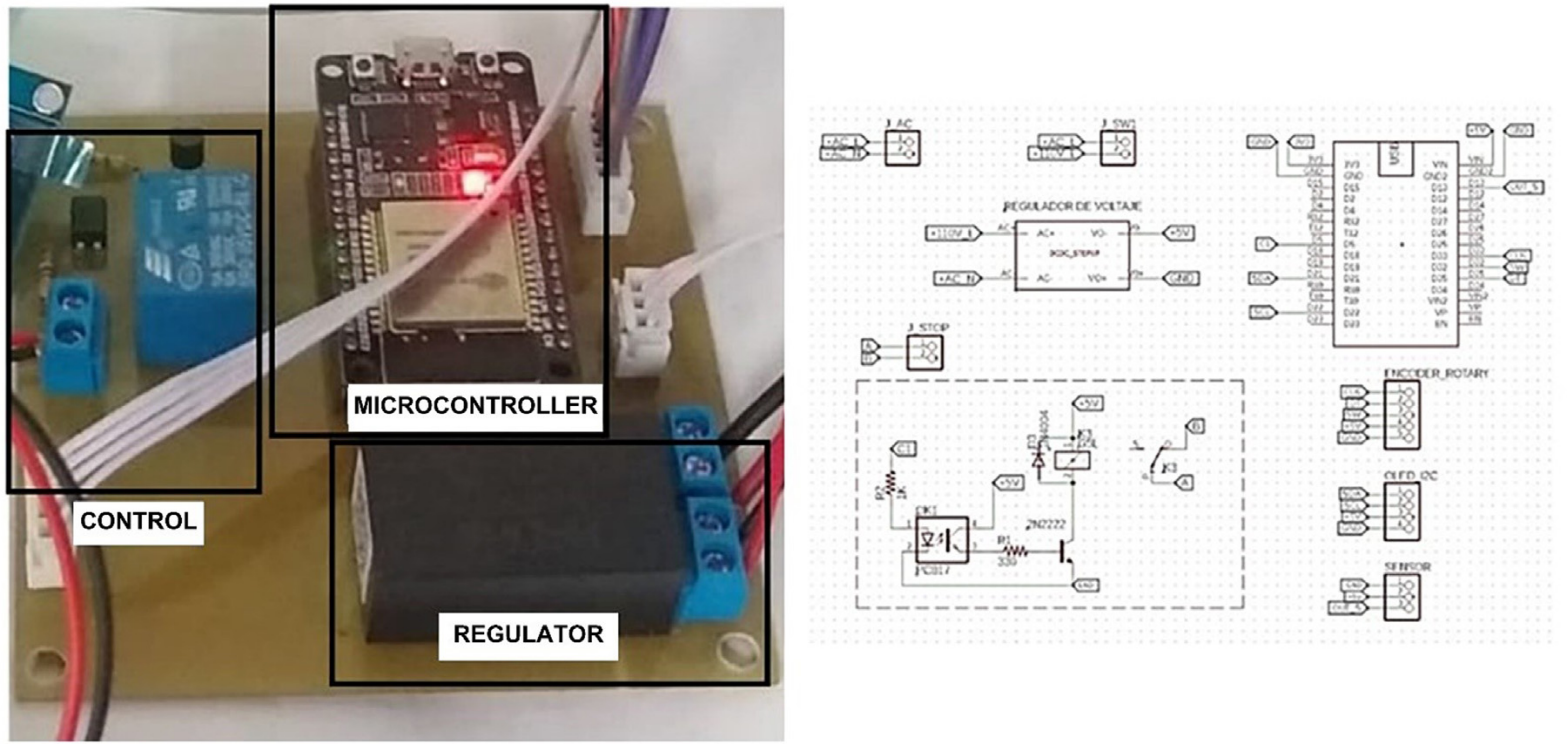
(a) Data acquisition mainboard, (b) electronic card schematic design, (c) electronic card routing view, and (d) manufactured electronic card.

This subsystem contains the power section, isolated by an optocoupler from the microcontroller. Activation of the mechanical relay, triggered by the signal from the optocoupler, opens the circuit of the motor shutdown button, sending a signal to the electric power controller to cease current flow to the equipment [24], as depicted in Fig. 18a.

#### Data acquisition mainboard

This subsystem serves as the core of the rotating control system for the equipment. It comprises two sections: hardware and firmware. The hardware includes a microcontroller, a rotational encoder for defining input parameters, digital outputs for an optocoupler, a mechanical relay for motor control, and a power system with a 110 V AC to 5 V DC converter. The dual-core microcontroller used, ESP32-WROOM-32S, communicates with the sensing, display, and control elements through programmed logic [25], as depicted in Fig. 18a. The design of the rotation control card was performed using Eagle Cadsoft software, academic version. This design phase involved the schematic design of the components (Fig. 18b) and routing that defines the physical locations of all elements: track size, spaces, footprint size, and other characteristics of each electronic component (Fig. 18c). The manufacturing of the printed circuit board (PCB, prototyping circuit board) was executed on a Bungard pro3000 CNC milling machine (Fig. 18d).

**Fig. 19:**
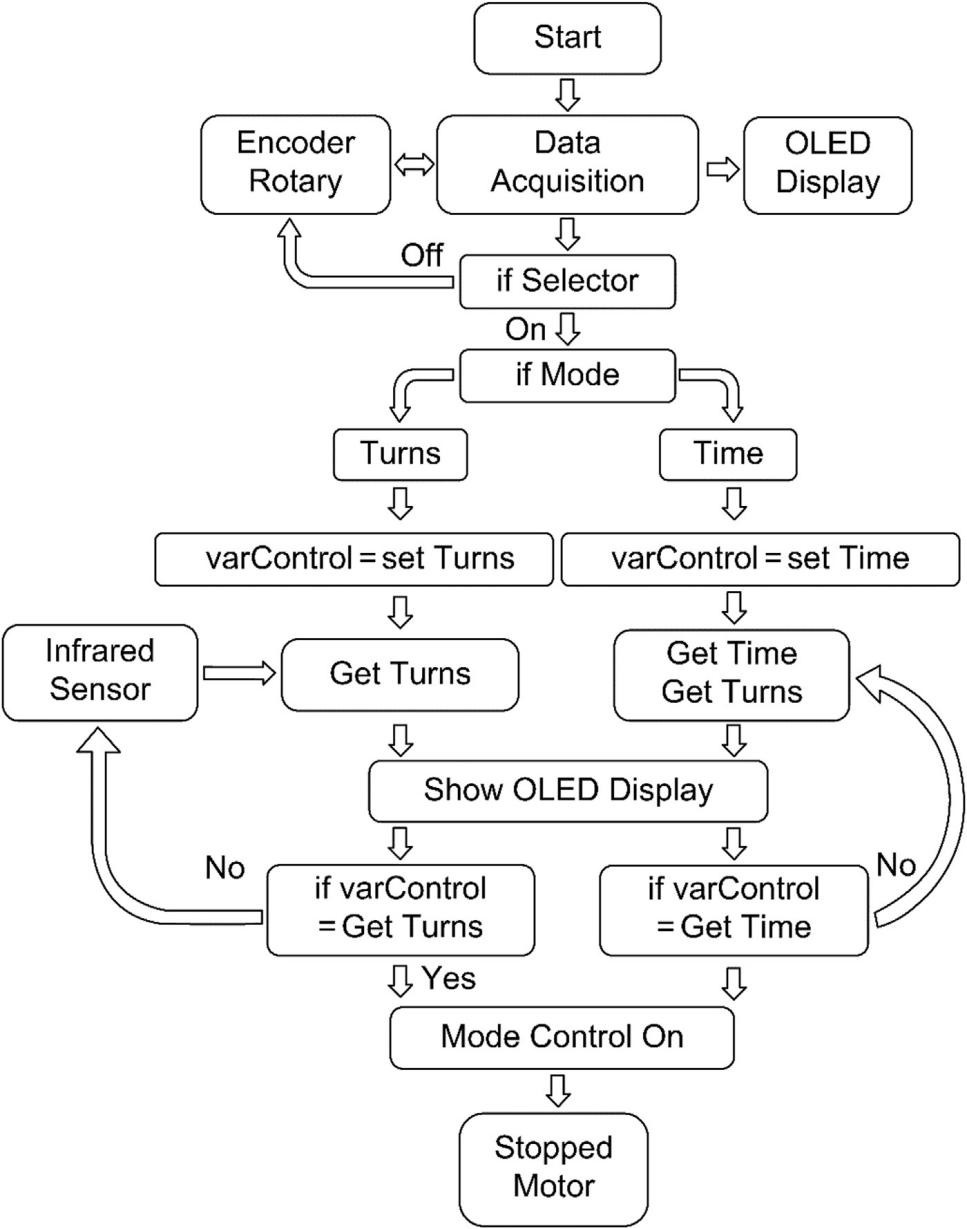
Firmware flowchart.

In the firmware, the microcontroller logic was developed to acquire data from the rotation encoder, which functions similarly to a potentiometer with an integrated button. The obtained signal enables the determination of the sensor rotation position, facilitating navigation through a menu displayed on the organic light-emitting diode (OLED) screen of the electronic system. This enables users to specify the number of rotations or the stop time of the equipment using the internal button as a selector. Additionally, data from the infrared sensor are acquired through general-purpose input/output pins to determine the number of rotations via a vane coupled to the equipment. When the number of rotations or time meets the user-defined parameters, the system signals to stop by opening the relay, effectively mimicking the action of digitally pressing the stop button (Fig. 19).

#### Stop control

**Fig. 20:**
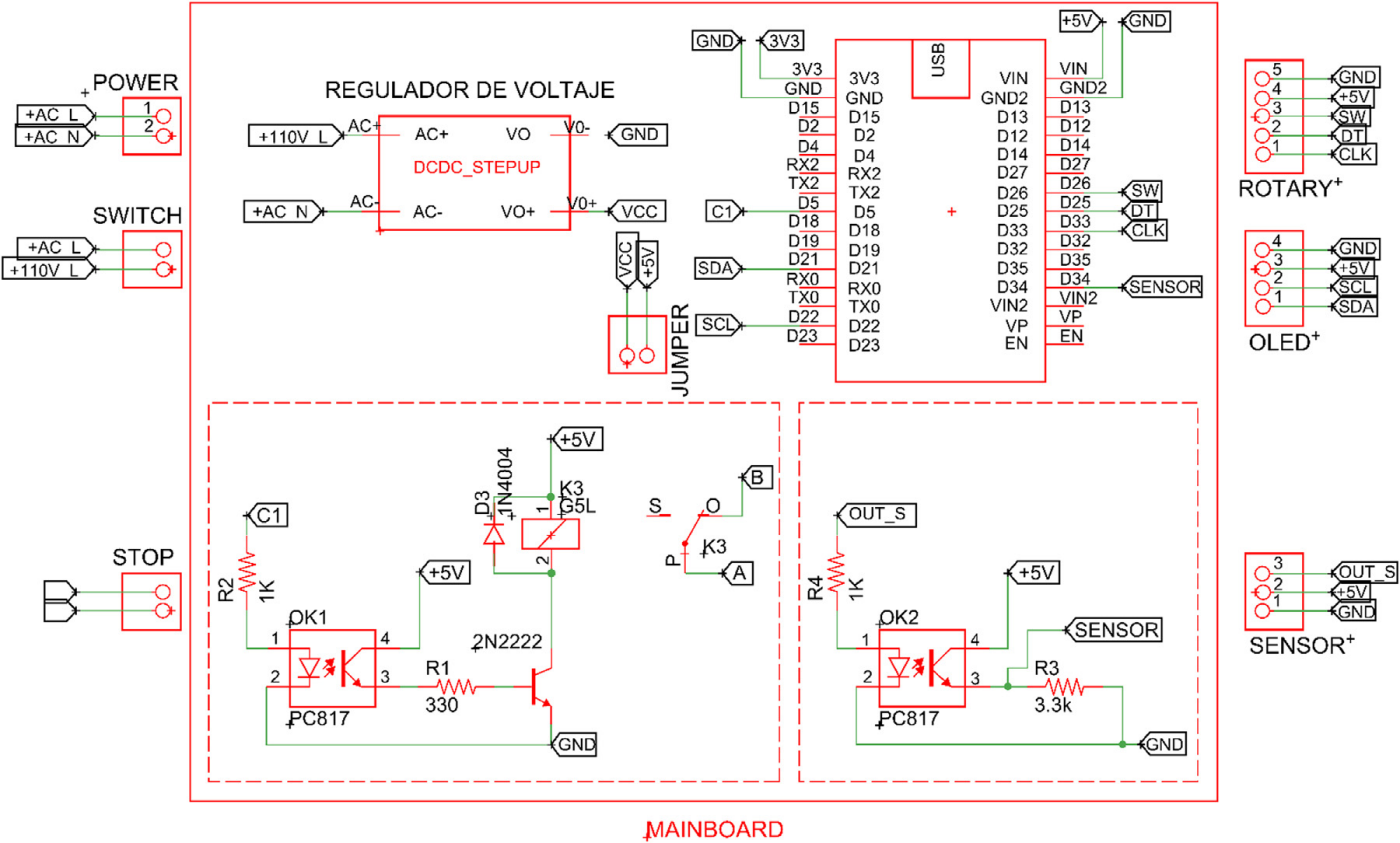
Wiring connection diagram.

In the general design of the equipment control board, the previously defined start and stop buttons were utilized. To enhance the control system, it was crucial to include an automatic stop feature based on the number of laps or the time specified by the user. Therefore, a layout of the elements that interact with the user was organized, including the card’s power switch, off button, rotary encoder, OLED display, and infrared sensor based on the connection diagram shown in Fig. 20.

#### Data visualization

**Fig. 21:**
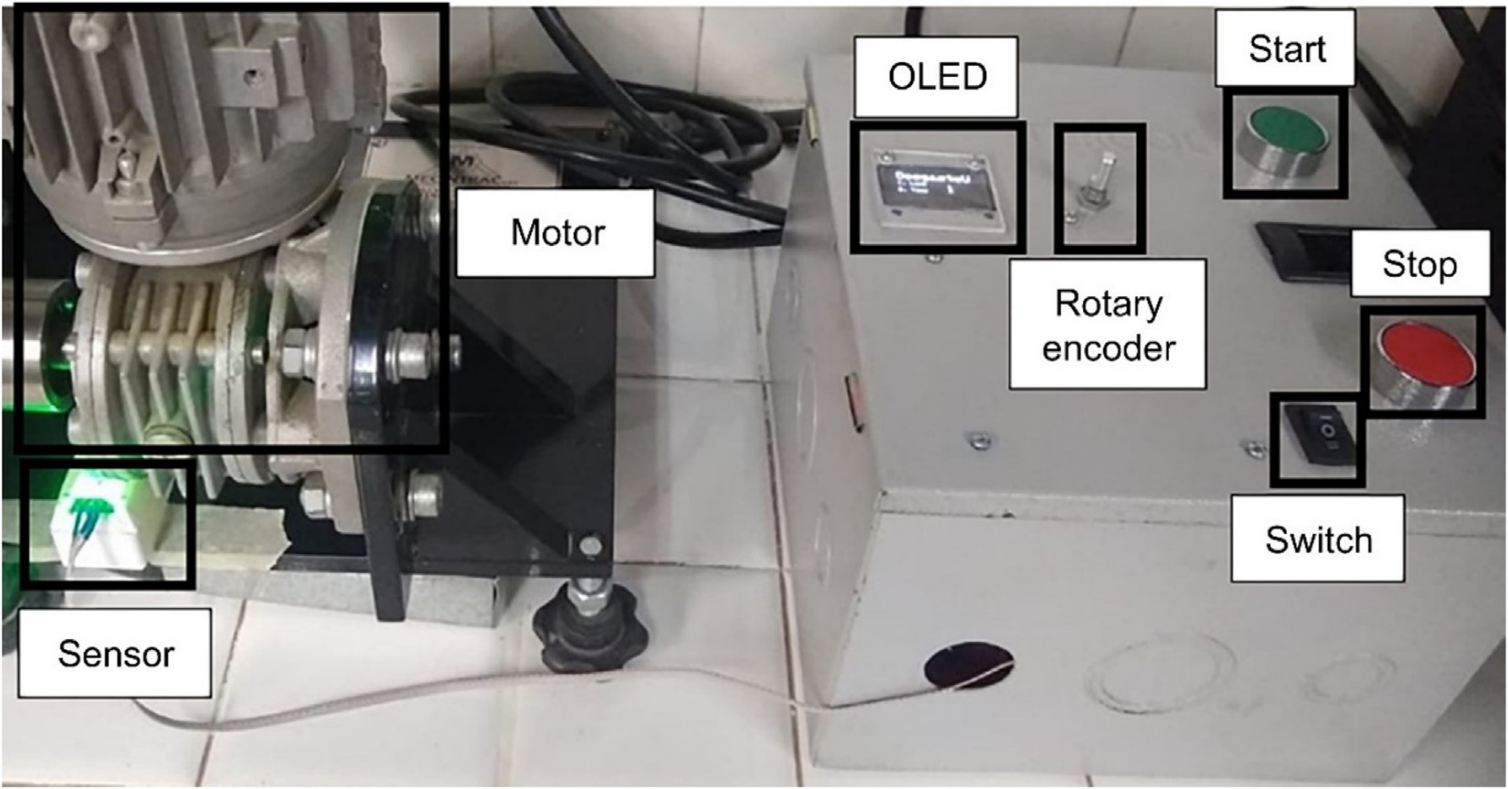
Mechatronic integration; control panel assembly view.

This subsystem features a 1.3ʺ OLED screen with I2C (inter-integrated circuit) communication from the microcontroller, displaying a menu of options for controlling the mode by number of rotations or time. Within each submenu, users can view the current number of rotations and/or elapsed time based on their selection. Additionally, it indicates whether the test has been initiated by the selector button using the “OK” option displayed on the screen, as illustrated in Fig. 21.

#### Mechatronic integration

In alignment with all the subsystems, the electronic card was integrated with the infrared sensing element. This involved docking the motor control system buttons, the power button card, and the installation of a rotary encoder and display screen on a conventional metal control panel (Fig. 21).

## Method validation

### Experimental tests and analysis

#### Control system

**Fig. 22:**
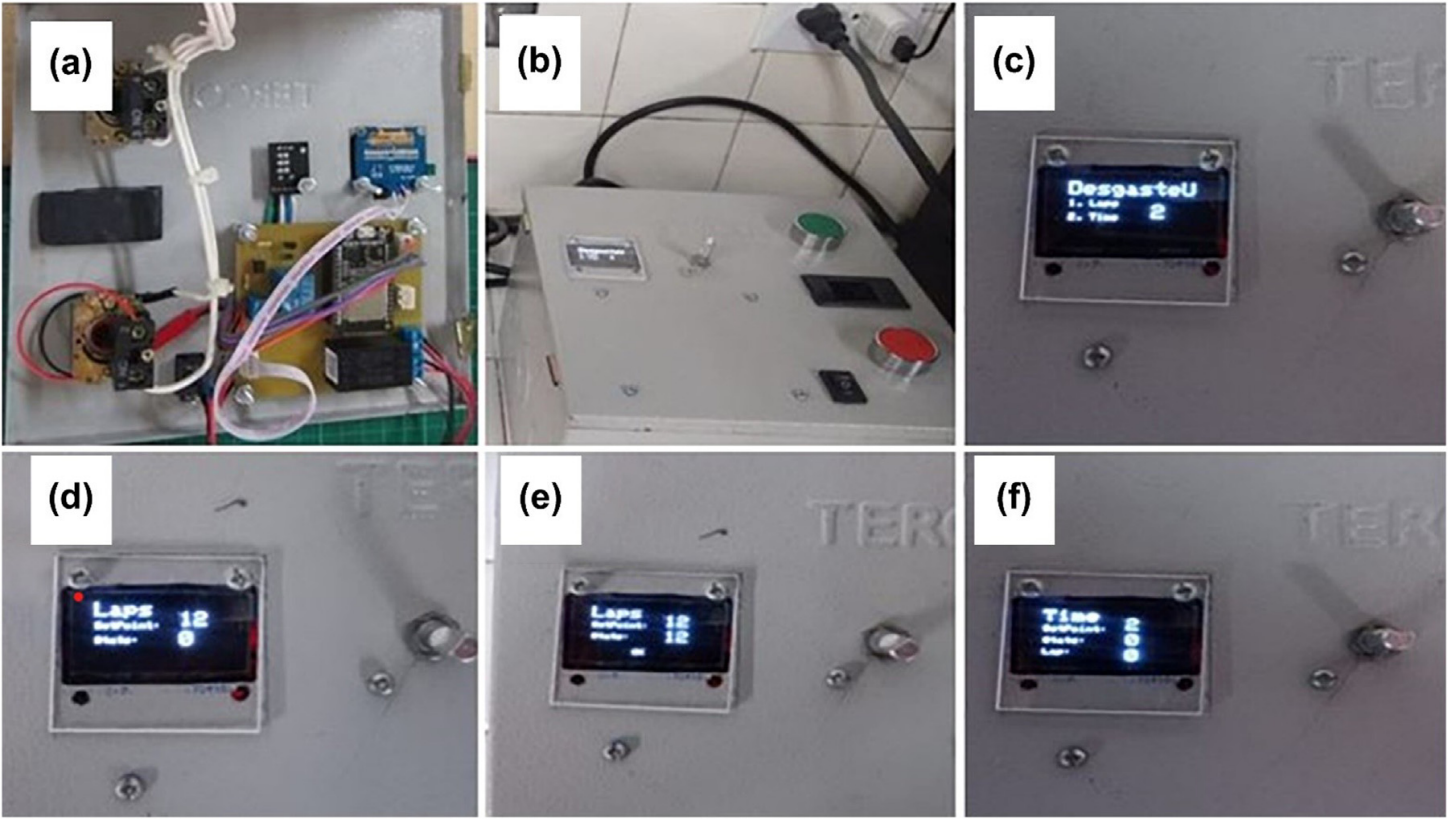
Steps for testing the control system: (a) electronic PCB, (b) control box, (c) main menu, (d) OLED display showing number of rotations reached, (e) OLED display indicating motor stopped by number of rotations, and (f) OLED screen indicating motor stopped by time.

The redesigned brushing equipment’s spin control system utilizes the ESP32 microcontroller, which is responsible for controlling the motor stoppage based on either the number of revolutions or time. Upon powering on the device, the main menu is displayed on the OLED screen, offering options to stop by rotations (“Laps: 1″) or time (“Time: 2”). Once the desired option is selected, another menu prompts the user to input the number of rotations or the duration of the motor shutdown. Pressing the green button initiates the test, with the OLED screen displaying rotation counts and/or elapsed time. Upon test completion, the relay state changes; the contactor is opened, and the electrical current is halted to turn off the motor. The procedure is as follows:

**Step 1.** The test point is defined during the installation of the electronic card within the equipment (Fig. 22a).

**Step 2**. The control box is connected to the 110 V AC outlet, after which the card is turned on using the black switch, and the start menu is displayed on the OLED screen (Fig. 22b).

**Step 3**. The main menu, “DegasteU,” is displayed, offering the options to stop by rotations (“Laps: 1″) or time (“Time: 2”), which are selected via the selector. Pressing down confirms the selection (Fig. 22c).

***“Laps” selection***

**Step 4.** With the selector, the number of rotations is chosen. The desired value of rotations is established in the test, and the word “OK” should appear on the screen (Fig. 22d).

**Step 5.** To start the motor, the green button is pressed, and the equipment starts to count the rotations; it can be stopped based on the user-defined value. The test is repeated ten times to verify the reliability of the equipment stop control system (Fig. 22e).

***“Time” selection***

**Step 6.** To enter the main menu, the selector is pressed, followed by searching for the number “0″; then, the selector is pressed again. If unsuccessful, the electronic card is reset. Subsequently, “Time” with option 2 is selected, and the equipment operation time is defined (Fig. 22f).

**Fig. 23:**
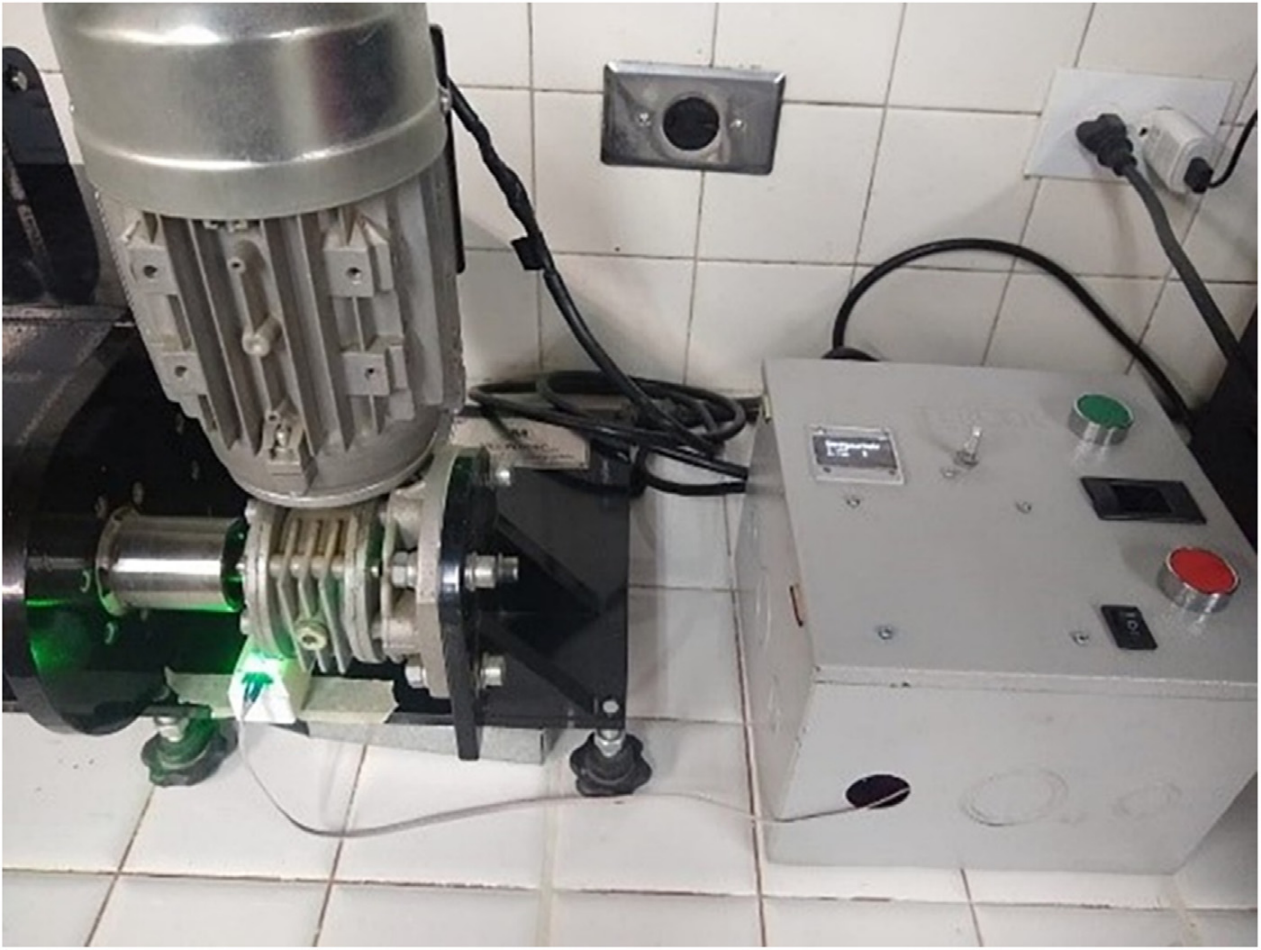
View of the prototype installed in operation.

**Step 7.** To start the motor, the green button is pressed. When the first rotation begins, the system initiates the timer. It is ensured that the sensor is correctly located with the sensing vane installed on the motor (Fig. 23).

#### Testing

Several control tests were conducted to validate the system based on the number of rotations (Table 2) and the maximum operating time (Table 3).

**Table 2:** Tests by user-defined number of rotations.

Test	Defined rotations	Rotations counted	State
1	12	12	Ok
2	10	10	Ok
3	30	30	Ok
4	7	7	Ok
5	10	10	Ok
6	8	8	Ok
7	11	11	Ok
8	30	30	Ok
9	60	60	Ok
10	120	120	Ok

**Table 3:** Tests by user-defined time.

Test	Defined time (min)	Measured time (min)	Rotations counted	State
1	2	2	120	Ok
2	2	2	120	Ok
3	3	3	180	Ok
4	3	3	180	Ok
5	2	2	120	Ok
6	4	4	240	Ok
7	4	4	240	Ok
8	2	2	120	Ok
9	2	2	120	Ok
10	2	2	120	Ok

The tests conducted by varying the number of rotations and the test duration yielded satisfactory results, with no deviations identified between the input variables and the response of the equipment. This validates that the control system effectively regulates the number of rotations and the downtime of the equipment. The control elements, combined with the built-in instrumentation, provide the equipment with reliability in test repeatability and acquired technological robustness. These process variables are fundamental operating parameters and were established as design objectives to meet both the technical requirements of the test and the needs specified by the user. The ability for users to view the current number of rotations and/or elapsed time based on their selection enables greater overall control of the test and the option to stop it at a specific moment according to an intrinsic need of the test or urgent circumstances.

In the configuration and operation of the instrumentation and control system, several features prove to be significant. The motor control system, equipped with a rotation sensor, allows for tests with user-defined parameters, such as the number of rotations or the defined test time. The rotational encoder controls equipment stoppages upon the completion of rotations or when the defined test time is reached. Additionally, the rotational encoder button enables parameter reconfiguration during the test and maintains the number of revolutions in case of an emergency stoppage. The OLED screen displays user-accessible menus for selecting the control mode —either by the number of rotations or time— and provides real-time information on test start status, rotations, and elapsed time. Finally, the electrical module operates adequately to protect the motor and ensure optimal energy supply for equipment startup and stoppage. It also features electrical isolation through an optocoupler that provides additional protection to the microcontroller.

#### Wear system

**Fig. 24:**
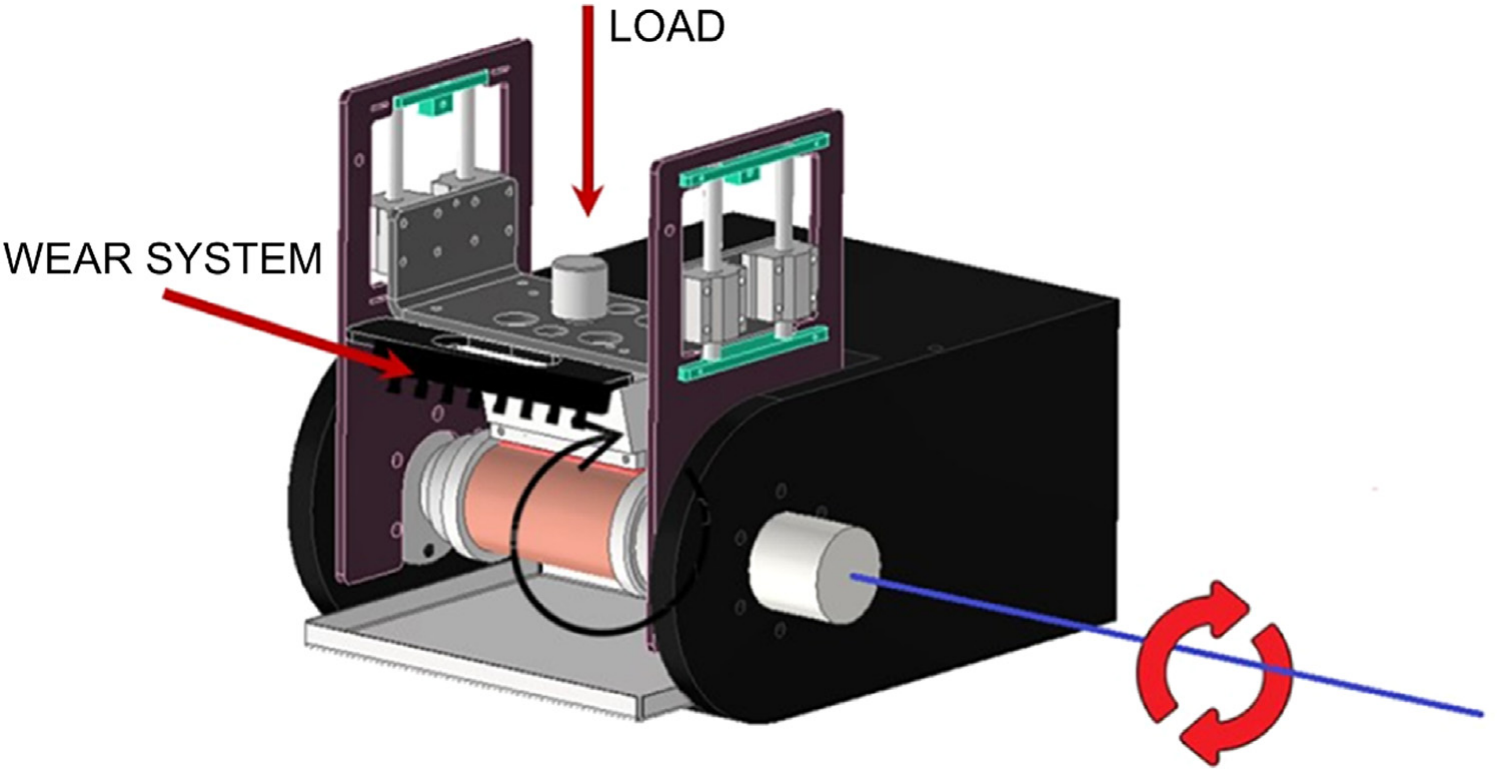
Illustration of wear test abrasion mechanism.

The basic principle of the test is to induce material loss from a sample through constant efforts and elements capable of causing abrasion to the material surface. The wear test simulates the continuous and progressive removal of material from a solid surface as a result of the mechanical interaction of two sliding surfaces under load, resulting in the consequent loss of mass and volume. During the test, the sample in contact with the wear system rotates at a specific speed while a load applies a radial force along its surface, as illustrated in Fig. 24. At the end of the test, the mass and volume loss of the sample are determined, providing information regarding the material’s wear resistance.

#### Testing

To evaluate the implemented wear systems, cylindrical specimens of natural soil with a diameter of 5 cm and a height of 10 cm were prepared and compacted. Table 4 presents the physicochemical properties of the soil.

**Table 4:** Soil properties.

Property	Value	Method
Soil classification (USCS)	CL	ASTM D2487
Soil classification (AASTHO)	A–7–6(13)*	ASTM D3282
Plasticity index ( %)	21	ASTM D4318
Maximum dry density (g/cm^3^)	1.626	ASTM D698
Optimum moisture content ( %)	22	ASTM D698
Sulfate content (mg SO_4_/g)	0.48	ASTM D516
pH at 22 °C	5.2	ASTM D4972

⁎ Clay soils with poor engineering properties; Group index 13: the soil has a low quality as a subgrade soil for a road.

The wear test methodology follows AASHTO T135 [6] and ASTM D559 [7] and it incorporates the model used by the Tanzania Bureau Standards [13] Furthermore, the methodology includes the use of alternative wear systems instead of the brush specified in the standard. The samples were weighed, clamped to the wear equipment with a load of 13 N (3 lbf), and then subjected to 60 revolutions. At the end of the test, the samples were weighed again, and the mass loss was determined as a percentage of the original mass. Several tests were conducted to assess the ability and strength of each wear system to impart abrasion to a material, and the results were compared with those obtained using the original brush. Table 5 presents the results of one of the tests, consisting of 4 cycles of 60 revolutions each.

**Table 5:** Wear test results for the three wear systems and original brush.

	Original brush			Serrated metal sheet			Metal brushes			240 grit sandpaper		
	Mass											
Cycle	Sample g	Loss		Sample g	Loss		Sample g	Loss		Sample g	Loss	
		g	%		g	%		g	%		g	%
0	330.01	–		326.81	–	–	323.12	–	–	314.52	–	–
1	327.01	3.00	0.91	320.20	6.61	2.02	321.70	1.42	0.44	313.81	0.71	0.23
2	324.91	2.10	0.64	311.38	8.82	2.75	320.30	1.40	0.44	313.01	0.80	0.25
3	323.21	1.70	0.52	302.65	8.73	2.80	319.18	1.12	0.35	312.39	0.62	0.20
4	321.93	1.28	0.40	296.07	6.58	2.17	318.18	1.00	0.31	311.78	0.61	0.19
Total		8.08	2.45	–	30.74	9.41	–	4.94	1.54	–	2.74	0.87

**Fig. 25:**
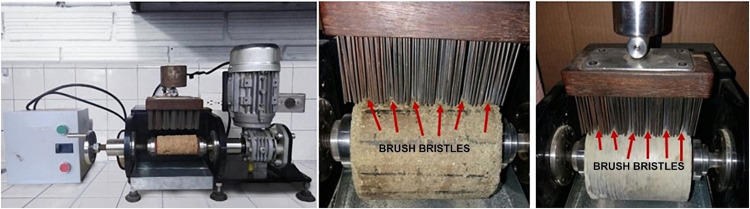
Original equipment and detail of the spacing between the brush bristles.

The results indicate that the serrated metal sheet system generates the most wear on the specimen, followed by the metal brushes system, and finally, the system that includes commercial sandpaper. Although the original brush is a mechanically robust element, this characteristic does not correlate directly with the expected mass loss, which had intermediate values compared to the other elements. The geometry of the brush is a limitation for the efficient operation of the wear equipment; the size and spacing between its bristles prevent them from contacting the entire surface of the specimen, leading to reduced contact as the specimen decreases in volume, an aspect that can be seen in Fig. 25. This circumstance also causes the mass loss to decrease rapidly, while in the other systems, it remains approximately constant during all cycles.

**Fig. 26:**
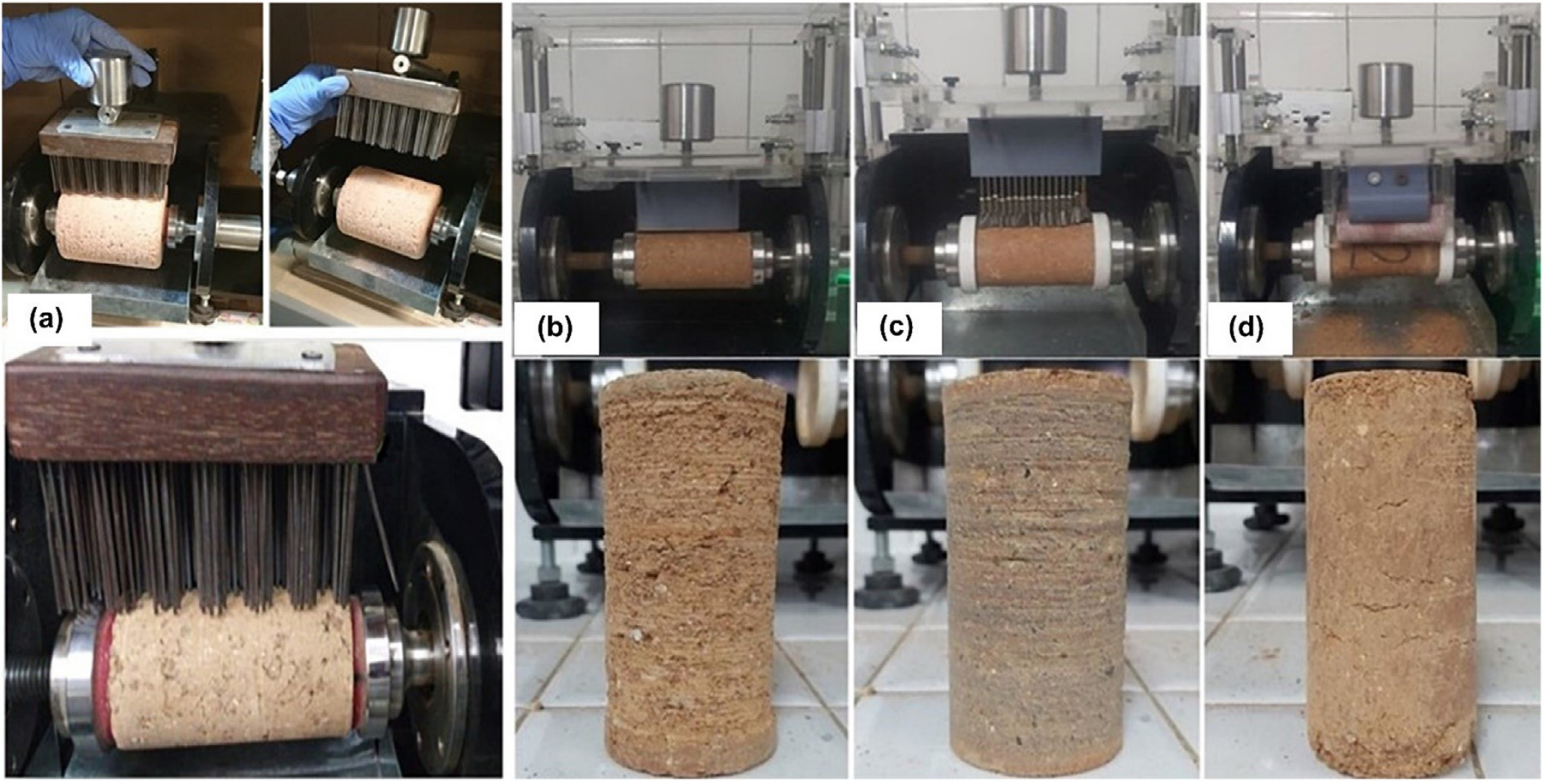
Final appearance of the specimens after the wear test using different wear elements: (a) original brush, (b) serrated metal sheet, (c) metal brushes, and (d) 240 grit sandpaper.

Concerning the performance of the different new wear systems used, the wear is much more uniform across the surface of the samples than that generated by the original brush. In this specimen, greater surface unevenness is observed, caused not only by the spaces between the brush bristles but also by the variation in the force applied through the pivoted arm with the decrease in the radius of the specimen during the test. Fig. 26 shows the final appearance of the specimens using different abrasive elements: original brush (Fig. 26a), serrated metal sheet (Fig. 26b), metal brushes (Fig. 26c), and 240 grit sandpaper (Fig. 26d).

## Limitations

None.

## CRediT authorship contribution statement

**Nathalia Marín-Pareja:** Conceptualization, Data curation, Investigation, Methodology, Validation, Visualization, Writing – original draft. **Camilo Rodríguez:** Methodology, Resources, Software, Validation, Visualization. **Eliana Llano:** Conceptualization, Data curation, Formal analysis, Investigation, Methodology, Visualization, Writing – original draft. **Gloria Restrepo:** Conceptualization, Formal analysis, Funding acquisition, Investigation, Methodology, Supervision, Validation, Visualization, Writing – original draft, Writing – review & editing.

## Declaration of competing interest

The authors declare that they have no known competing financial interests or personal relationships that could have appeared to influence the work reported in this paper.

## Data Availability

Not applicable
